# Comparative transcriptomics reveals the conserved building blocks involved in parallel evolution of diverse phenotypic traits in ants

**DOI:** 10.1186/s13059-016-0902-7

**Published:** 2016-03-07

**Authors:** Claire Morandin, Mandy M. Y. Tin, Sílvia Abril, Crisanto Gómez, Luigi Pontieri, Morten Schiøtt, Liselotte Sundström, Kazuki Tsuji, Jes Søe Pedersen, Heikki Helanterä, Alexander S. Mikheyev

**Affiliations:** Centre of Excellence in Biological Interactions, Department of Biological and Environmental Sciences, University of Helsinki, Helsinki, Finland; Tvärminne Zoological Station, University of Helsinki, J.A. Palménin tie 260, FI-10900 Hanko, Finland; Okinawa Institute of Science and Technology, 1919-1 Tancha Onna-son, Kunigami-gun, Okinawa 904-0412 Japan; Department of Environmental Sciences, University of Girona, Campus Montilivi, 17071 Girona, Spain; Centre for Social Evolution, Department of Biology, University of Copenhagen, Universitetsparken 15, DK-2100 Copenhagen, Denmark; Department of Subtropical Agro-Environmental Sciences, University of the Ryukyus, Senbaru 1, Nishihara, Okinawa 903-0213 Japan; Research School of Biology, Australian National University, Canberra, ACT 0200 Australia

**Keywords:** Social insects, Caste differentiation, Gene expression, Parallel evolution, Phenotypic plasticity, Gene co-expression network

## Abstract

**Background:**

Reproductive division of labor in eusocial insects is a striking example of a shared genetic background giving rise to alternative phenotypes, namely queen and worker castes. Queen and worker phenotypes play major roles in the evolution of eusocial insects. Their behavior, morphology and physiology underpin many ecologically relevant colony-level traits, which evolved in parallel in multiple species.

**Results:**

Using queen and worker transcriptomic data from 16 ant species we tested the hypothesis that conserved sets of genes are involved in ant reproductive division of labor. We further hypothesized that such sets of genes should also be involved in the parallel evolution of other key traits. We applied weighted gene co-expression network analysis, which clusters co-expressed genes into modules, whose expression levels can be summarized by their ‘eigengenes’. Eigengenes of most modules were correlated with phenotypic differentiation between queens and workers. Furthermore, eigengenes of some modules were correlated with repeated evolution of key phenotypes such as complete worker sterility, the number of queens per colony, and even invasiveness. Finally, connectivity and expression levels of genes within the co-expressed network were strongly associated with the strength of selection. Although caste-associated sets of genes evolve faster than non-caste-associated, we found no evidence for queen- or worker-associated co-expressed genes evolving faster than one another.

**Conclusions:**

These results identify conserved functionally important genomic units that likely serve as building blocks of phenotypic innovation, and allow the remarkable breadth of parallel evolution seen in ants, and possibly other eusocial insects as well.

**Electronic supplementary material:**

The online version of this article (doi:10.1186/s13059-016-0902-7) contains supplementary material, which is available to authorized users.

## Background

Understanding how novel phenotypes arise and are maintained is a major goal of evolutionary biology [1–3]. Parallel evolution of conserved sets of genes across related species may lead to parallel appearances of phenotypes in response to similar selective regimes [4, 5] (e.g., antibiotic resistance [6], nacre building in molluscs [7]). Redeployment of pre-existing genes and pathways permits the parallel evolution of phenotypic novelty [5, 8–10]. Furthermore, novel phenotypes frequently arise through functional changes in conserved developmental pathways in closely related species (e.g., the Wnt signaling pathway [11], wing pigmentation in butterflies [12, 13]). It is well recognized that most genes act as members of biological pathways, or of co-regulated modules [14], yet how gene networks evolve and to what extent they play a role in the origin of phenotypic novelty remain unsolved [15].

Reproductive division of labor in social insects provides an extreme example of phenotypic plasticity, in which a single totipotent egg may develop into either a reproductive queen or a non-reproductive worker [16]. As a result, eusocial insects decouple behavioral and physiological traits into these two complementary phenotypes, called castes. Eusociality is one of the major transitions in evolution [17], and has arisen independently multiple times in the order Hymenoptera, comprising wasps, bees, and ants [18]. Building on the evolution of eusociality, queen–worker polymorphisms have also evolved independently in several lineages. To understand the molecular mechanisms underlying social insect polymorphism, previous studies have primarily examined caste-biased gene expression patterns in a small number of distantly related species, typically across separate origins of eusociality [19–25]. These studies have found a small number of genes repeatedly associated with reproductive division of labor, but a comprehensive, comparative characterization of queen and worker transcriptional architecture has been lacking.

Evolution of reproductive division of labor in social insects has interested evolutionary biologists since Darwin. Sterile workers cannot directly transmit traits they possess [26, 27], but worker phenotypes respond to natural selection indirectly, through the action of kin selection, giving rise to a diverse array of morphological and behavioral adaptations. Despite the low overlap across eusocial species in the number of consistently caste-biased genes, the common assumption that castes have distinct transcriptional profiles has motivated a wide range of studies examining patterns of selection acting on queen versus worker protein sequences, which are believed to exist in separate selective environments [26, 27]. Recent studies have shown that genes with caste-biased expression evolve faster at the sequence level than their non-biased counterparts, although the detected direction of selection for queen- and worker-biased genes was sometimes opposite [28, 29]. However, other factors may also affect the strength of selection acting on caste-biased genes. Some of them are directly linked to social insect life histories, such as an increased number of queens per colony, which is predicted to affect the strength of selection acting on the worker genes by weakening relatedness within colony [27]. Also, based on extensive data from a wide variety of species (ranging from yeast [30] to mammals [31]), we know that evolution of genes is shaped to a large extent by their levels of expression and interactivity with other genes [32]. More recently, this has also been confirmed in social insects [33, 34]. However, the regulatory architecture that governs queen and worker phenotypes remains largely unknown in social insects. Thus, inferring the global regulatory environment of caste-biased genes is the key to understanding their long-term evolution.

The idea of a shared “genetic toolkit” across the several eusocial lineages in Hymenoptera is based on the Evo-Devo conceptual framework, which has shown that convergent use of conserved sets of genes is often involved in animal development and morphological innovation [20, 21, 35–37]. A recent study [24] identified only a small number of genes (15) constantly differentially expressed across three distantly related hymenopteran species, but more overlap at the level of pathway and biological function (five KEGG (Kyoto Encyclopedia of Genes and Genomes) pathways, five enriched Gene Ontology (GO) functional categories).

In the current study, we take a novel approach to study caste differentiation, and use weighted gene co-expression network analysis (WGCNA) to define conserved sets of co-regulated genes underlying queen and worker phenotypic traits, and other ant phenotypic traits. WGCNA analysis provides an overview of the transcriptomic organization [38, 39], and the relationships between sets of genes with external, biological traits [40]. This is a more complex approach than traditional pairwise differential gene expression since it takes into consideration the relationships between genes via pairwise correlations between gene expression profiles. WGCNA allows the identification of modules of co-expressed genes constructed from the expression profiles of all individuals simultaneously by using a hierarchical clustering approach. This step operates on all data simultaneously and does not require any a priori information about the biological source of sequenced libraries (e.g., which were made from queens and which from workers). Instead, after constructing the gene modules, each module global expression profile can be correlated with external traits to look for significant associations [40].

Whereas most previous studies have focused on examining whether the same genes were involved across origins of eusociality, we focus on ants, an ecologically diverse group sharing the same origin of eusociality. First, we test whether conserved sets of genes are involved in queen/worker phenotypic differentiation. Second, we test whether these genes are also involved in the parallel evolution of other species-level traits. Third, we test predictions that non-caste-associated and caste-associated sets of co-expressed genes evolve at different rates by taking into account some of the network properties. Because the investigation focuses on the adult stage, our data are not suitable for testing the developmental toolkit hypothesis, which has been the primary focus of investigation in social insects [20, 21]. However, the overall question is conceptually the same: are conserved regulatory modules involved in reproductive division of labor?

**Fig. 1:**
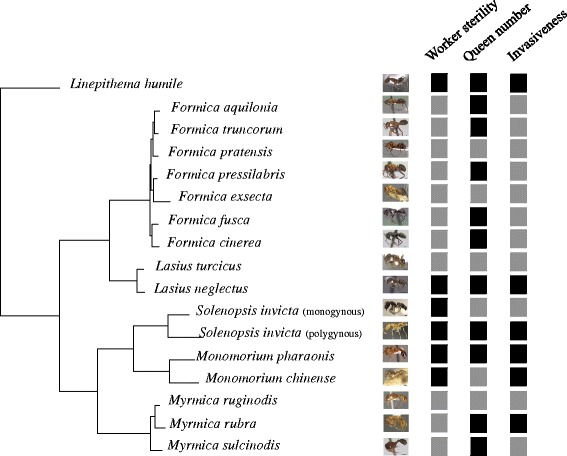
Phylogenetic relationships of 16 ant species studied shown with their pictures (source http://www.antweb.org/) and three biological traits: worker sterility (*grey square*, can lay unfertilized eggs; *black square*, completely sterile), colony queen number (*grey square*, single queen; *black square*, multiple queens), and invasiveness (*grey square*, not invasive; *black square*, can be invasive). The phylogenetic tree was constructed using OGG alignments with the software RAxML (v. 8) [87]. The data set contained 1427 genes and 3.59 Mb of sequence, and the analysis was partitioned by gene and conducted under a GTRGAMMAI model

We identified common transcriptional profiles in female castes (queens and workers) from 16 ant species (including two social forms of *Solenopsis invicta*) from three subfamilies, which differed in a variety of key traits (Fig. 1). We found that connectivity, expression levels and their interactions were strongly correlated with evolutionary rates of protein coding genes. The inferred modules are involved in caste phenotypes and other derived traits important to social evolution, such as complete worker sterility, the number of queens per colony, and even the ecological invasiveness of a species. These results suggest that evolutionarily stable modular genetic networks participate in phenotypic maintenance of reproductive division of labor. However, in addition to caste differentiation, these modules play other roles, and parallel co-option of these regulatory building blocks may also result in repeated evolution of complex phenotypes.

## Results

### Assembly of transcriptomes

We sequenced 100 libraries (50 each from queens and workers), representing three biological replicates of each caste (two replicates for *Formica exsecta* [19]), using whole-body samples. We recovered 719 Gb (on average 42 Gb per species) of 100-bp paired-end reads. Following quality filtering, we constructed a de novo transcriptome for each species separately using Trinity (release 2013-02-25 [41, 42]). The initial transcriptomes had a total assembly length between 87.4 Mb and 620.8 Mb, and the number of contigs varied between 77,922 and 161,555. The transcriptome contigs were cleaned from probable exogenous RNAs and only contigs that showed a significant BLAST hit to at least one of the nine published hymenopteran genomes (seven ant species, *Apis mellifera*, and *Nasonia vitripennis*) were kept for further analysis (on average 60 % of the contigs), thus providing evidence that the contigs are “true” genes and not sequencing or assembly artifacts. De novo assembly enables functional genomic studies, but has the potential for mis-assemblies [43] and consequent biases in downstream analyses. Also, we focused on the presence of conserved co-expressed sets of genes, and not on the presence of taxonomically restricted genes, which are not likely to be conserved across related species. We made the choice to focus on “true” genes rather than unconfirmed contig expression patterns. The initial and final number of contigs can be found in Additional file 1. After these quality-filtering steps, the final Trinity assemblies contained between 26,666 (*Myrmica sulcinodis*) and 77,633 contigs (*F. exsecta*), with an average number of 44,171 contigs. In total, 167,918 transcripts from the 16 species were assigned to 9859 orthologous gene groups (OGGs).

### Constructing the ant gene co-expression network for all 16 species

**Fig. 2:**
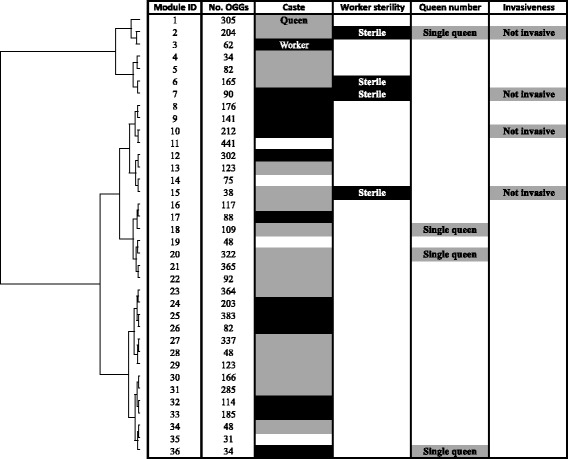
Correlation between module eigengenes and the biological traits (caste, worker sterility, colony queen number and invasiveness). Modules were clustered based on GO term similarities obtained with GOSemSim [93], which computes semantic similarity among sets of GO terms (Additional file 8). Expression of most modules is strongly associated with caste phenotypes. In addition, expression of several of these modules was also associated with other phenotypes, such as obligate worker sterility, colony queen number, and invasiveness. This shows that modules likely play multiple roles, and that their constituent genes have many functions

We used the WGCNA package [40] to construct a weighted gene co-expression network analysis (WGNCA) on the entire data set using the mean of normalized expression counts for each OGG. WGCNA takes correlations between gene expression patterns across sequenced libraries and aggregates genes with similar profiles into ‘modules’. In addition to reducing the dimensionality of data in this manner, a gene co-expression network also describes connections between genes, which can be used to study their possible interactions and network-level properties [39, 44]. A total of 9859 OGGs of expression data from all 16 species were analyzed with the WGCNA package [40]. The input dataset consists of a table with each row representing one of the 9859 OGGs and each column one of the 100 samples (Additional file 2). Modules of co-expressed genes are inferred using the expression profiles of each sample regardless of the species and caste. After the cleaning step, 2432 OGGs were subsequently removed from the calculation owing to too many missing samples or zero variance, which may affect our ability to detect gene co-expression (Additional file 3). After merging modules of highly co-expressed OGGs, the final co-expression networks comprised 36 modules with >30 OGGs with an average number of 206 (standard deviation 118) (Fig. 2). A total of 5989 OGGs (75 % of the total number of OGGs) initially analyzed were assigned to co-expressed modules, and each module contains expression data from all 16 species.

An online resource has been created to simplify visualization of module organization, particularly the roles of key genes (the website is available at http://mikheyevlab.github.io/Comparative-transcriptomics-of-ants/). The online tool allows users to browse each module individually and to visualize expression levels and interactions between key genes directly.

### Conserved gene co-expression modules correspond to female caste traits

We calculated the eigengene, which is a single value for each sample for each module representative of the gene expression profiles of the samples in a module. The extent of module involvement in various biological processes can be tested by correlating eigengenes with external traits, such as phenotypes [38, 40]. To test the hypothesis that conserved sets of genes are involved in queen/worker phenotypic differentiation, we investigated the relationship between the eigengenes and the caste phenotypic traits, while controlling for any phylogenetic bias in the dataset, using a phylogenetic mixed model implemented in the MCMCglmm package [45]. Expression of 32 out of 36 modules was significantly correlated with one of the two female castes (Fig. 2; Additional file 4; worker caste, 13; queen caste, 19). WGCNA does not use information on caste-specific differential gene expression when inferring modules, so modules that correlated strongly with caste were inferred by WGCNA without a priori knowledge about caste-biased expression patterns, and include both caste-biased and non-caste-biased genes (Additional file 5). Each module represents a set of co-expressed (and presumably interacting) genes [46] that has been conserved across the ant phylogeny (Fig. 2; Additional file 6).

To gain insight into the biological relevance and functional significance of modules, we performed GO enrichment analysis on the OGGs in each module. Complete lists of BLAST annotations and GO terms associated with each module are available in Additional files 7 and 8. The modules are enriched for specific biological functions related to either worker or queen phenotypes. For instance, two worker-associated modules (7 and 10) are linked to behavior and sensory perception, in accordance with what we would expect from their life history traits (Table 1; Additional file 8) [47].

**Table 1:** GO terms found enriched in each module

Module	Caste	Worker sterility	Queen number	Invasiveness	GO Term
1	Queen	NTA	NTA	NTA	cellular protein modification process
1	Queen	NTA	NTA	NTA	protein modification process
1	Queen	NTA	NTA	NTA	macromolecule modification
1	Queen	NTA	NTA	NTA	cellular protein metabolic process
1	Queen	NTA	NTA	NTA	cytoplasm organization
2	Queen	Sterile	Single	Not invasive	positive regulation of actin nucleation
2	Queen	Sterile	Single	Not invasive	positive regulation of Arp2/3 complex-mediated actin nucleation
2	Queen	Sterile	Single	Not invasive	regulation of Arp2/3 complex-mediated actin nucleation
2	Queen	Sterile	Single	Not invasive	phosphorylation
2	Queen	Sterile	Single	Not invasive	protein metabolic process
4	Queen	NTA	NTA	NTA	negative regulation of Ras protein signal transduction
4	Queen	NTA	NTA	NTA	negative regulation of small GTPase mediated signal transduction
4	Queen	NTA	NTA	NTA	negative regulation of signal transduction
4	Queen	NTA	NTA	NTA	Ras protein signal transduction
4	Queen	NTA	NTA	NTA	negative regulation of response to stimulus
5	Queen	NTA	NTA	NTA	cellular response to alcohol
5	Queen	NTA	NTA	NTA	adenine salvage
5	Queen	NTA	NTA	NTA	cellular response to ecdysone
5	Queen	NTA	NTA	NTA	regulation of protein secretion
5	Queen	NTA	NTA	NTA	negative regulation of protein secretion
6	Queen	Sterile	NTA	NTA	mitotic DNA damage checkpoint
6	Queen	Sterile	NTA	NTA	mitotic DNA integrity checkpoint
6	Queen	Sterile	NTA	NTA	regulation of protein ubiquitination
6	Queen	Sterile	NTA	NTA	negative regulation of protein ubiquitination
6	Queen	Sterile	NTA	NTA	positive regulation of protein ubiquitination
7	Worker	Sterile	NTA	Not invasive	autophagy
7	Worker	Sterile	NTA	Not invasive	G-protein coupled receptor signaling pathway
7	Worker	Sterile	NTA	Not invasive	regulation of synaptic transmission, cholinergic
7	Worker	Sterile	NTA	Not invasive	selenocysteinyl-tRNA(Sec) biosynthetic process
7	Worker	Sterile	NTA	Not invasive	intraspecies interaction between organisms
8	Worker	NTA	NTA	NTA	protein import into mitochondrial matrix
8	Worker	NTA	NTA	NTA	negative regulation of TOR signaling
8	Worker	NTA	NTA	NTA	water-soluble vitamin metabolic process
8	Worker	NTA	NTA	NTA	cellular aldehyde metabolic process
8	Worker	NTA	NTA	NTA	vitamin metabolic process
9	Worker	NTA	NTA	NTA	double-strand break repair
9	Worker	NTA	NTA	NTA	sphingolipid metabolic process
9	Worker	NTA	NTA	NTA	double-strand break repair via homologous recombination
9	Worker	NTA	NTA	NTA	recombinational repair
9	Worker	NTA	NTA	NTA	sodium ion transport
10	Worker	NTA	NTA	Not invasive	system process
10	Worker	NTA	NTA	Not invasive	neurological system process
10	Worker	NTA	NTA	Not invasive	sensory perception of chemical stimulus
10	Worker	NTA	NTA	Not invasive	sensory perception
10	Worker	NTA	NTA	Not invasive	locomotory behavior
11	NTA	NTA	NTA	NTA	cell division
11	NTA	NTA	NTA	NTA	macromolecule metabolic process
11	NTA	NTA	NTA	NTA	negative regulation of developmental process
11	NTA	NTA	NTA	NTA	DNA conformation change
11	NTA	NTA	NTA	NTA	cellular component biogenesis
12	Worker	NTA	NTA	NTA	myofibril assembly
12	Worker	NTA	NTA	NTA	dicarboxylic acid metabolic process
12	Worker	NTA	NTA	NTA	carbohydrate metabolic process
12	Worker	NTA	NTA	NTA	actomyosin structure organization
12	Worker	NTA	NTA	NTA	striated muscle cell development
13	Queen	NTA	NTA	NTA	RNA 3'-end processing
13	Queen	NTA	NTA	NTA	melanin biosynthetic process
13	Queen	NTA	NTA	NTA	snRNA 3'-end processing
13	Queen	NTA	NTA	NTA	U6 snRNA 3'-end processing
13	Queen	NTA	NTA	NTA	mRNA polyadenylation
14	NTA	NTA	NTA	NTA	receptor clustering
14	NTA	NTA	NTA	NTA	spinal cord development
14	NTA	NTA	NTA	NTA	peptide metabolic process
14	NTA	NTA	NTA	NTA	cellular amide metabolic process
14	NTA	NTA	NTA	NTA	neuromuscular synaptic transmission
15	Queen	Sterile	NTA	Not invasive	proteolysis
15	Queen	Sterile	NTA	Not invasive	Notch signaling pathway
15	Queen	Sterile	NTA	Not invasive	ephrin receptor signaling pathway
15	Queen	Sterile	NTA	Not invasive	establishment of body hair or bristle planar orientation
15	Queen	Sterile	NTA	Not invasive	lipid transport
16	Queen	NTA	NTA	NTA	leukocyte differentiation
16	Queen	NTA	NTA	NTA	in utero embryonic development
16	Queen	NTA	NTA	NTA	neural precursor cell proliferation
16	Queen	NTA	NTA	NTA	stem cell proliferation
16	Queen	NTA	NTA	NTA	chordate embryonic development
17	Worker	NTA	NTA	NTA	chitin metabolic process
17	Worker	NTA	NTA	NTA	amino sugar metabolic process
17	Worker	NTA	NTA	NTA	glucosamine-containing compound metabolic process
17	Worker	NTA	NTA	NTA	aminoglycan metabolic process
17	Worker	NTA	NTA	NTA	carbohydrate derivative metabolic process
18	Queen	NTA	Single	NTA	cellular transition metal ion homeostasis
18	Queen	NTA	Single	NTA	transition metal ion homeostasis
18	Queen	NTA	Single	NTA	DNA topological change
18	Queen	NTA	Single	NTA	transition metal ion transport
18	Queen	NTA	Single	NTA	snRNA metabolic process
19	NTA	NTA	NTA	NTA	one-carbon compound transport
19	NTA	NTA	NTA	NTA	urea transport
19	NTA	NTA	NTA	NTA	tRNA 5'-leader removal
19	NTA	NTA	NTA	NTA	urea transmembrane transport
19	NTA	NTA	NTA	NTA	cellular macromolecule localization
20	Queen	NTA	Single	NTA	nuclear-transcribed mRNA catabolic process, nonsense-mediated decay
20	Queen	NTA	Single	NTA	cellular macromolecule catabolic process
20	Queen	NTA	Single	NTA	cellular localization
20	Queen	NTA	Single	NTA	tissue regeneration
20	Queen	NTA	Single	NTA	nuclear export
21	Queen	NTA	NTA	NTA	Rho protein signal transduction
21	Queen	NTA	NTA	NTA	Ras protein signal transduction
21	Queen	NTA	NTA	NTA	cell adhesion
21	Queen	NTA	NTA	NTA	biological adhesion
21	Queen	NTA	NTA	NTA	regulation of Ras protein signal transduction
22	Queen	NTA	NTA	NTA	cellular protein metabolic process
22	Queen	NTA	NTA	NTA	single-organism intracellular transport
22	Queen	NTA	NTA	NTA	protein metabolic process
22	Queen	NTA	NTA	NTA	autophagic cell death
22	Queen	NTA	NTA	NTA	salivary gland cell autophagic cell death
23	Queen	NTA	NTA	NTA	purine ribonucleoside catabolic process
23	Queen	NTA	NTA	NTA	ribonucleoside catabolic process
23	Queen	NTA	NTA	NTA	purine nucleotide catabolic process
23	Queen	NTA	NTA	NTA	purine nucleoside catabolic process
23	Queen	NTA	NTA	NTA	nucleoside catabolic process
24	Worker	NTA	NTA	NTA	cellular amino acid metabolic process
24	Worker	NTA	NTA	NTA	positive regulation of cysteine-type endopeptidase activity involved in apoptotic process
24	Worker	NTA	NTA	NTA	alcohol catabolic process
24	Worker	NTA	NTA	NTA	positive regulation of cysteine-type endopeptidase activity
24	Worker	NTA	NTA	NTA	positive regulation of endopeptidase activity
25	Worker	NTA	NTA	NTA	cofactor metabolic process
25	Worker	NTA	NTA	NTA	cofactor biosynthetic process
25	Worker	NTA	NTA	NTA	single-organism biosynthetic process
25	Worker	NTA	NTA	NTA	coenzyme metabolic process
25	Worker	NTA	NTA	NTA	coenzyme biosynthetic process
26	Worker	NTA	NTA	NTA	ribosome assembly
26	Worker	NTA	NTA	NTA	organophosphate catabolic process
26	Worker	NTA	NTA	NTA	carbohydrate derivative catabolic process
26	Worker	NTA	NTA	NTA	extracellular polysaccharide metabolic process
26	Worker	NTA	NTA	NTA	extracellular polysaccharide biosynthetic process
27	Worker	NTA	NTA	NTA	cellular metabolic process
27	Queen	NTA	NTA	NTA	DNA metabolic process
27	Queen	NTA	NTA	NTA	cellular process
27	Queen	NTA	NTA	NTA	cellular macromolecule metabolic process
27	Queen	NTA	NTA	NTA	nucleobase-containing compound metabolic process
28	Queen	NTA	NTA	NTA	primary metabolic process
28	Queen	NTA	NTA	NTA	organic substance metabolic process
28	Queen	NTA	NTA	NTA	protein folding
28	Queen	NTA	NTA	NTA	cellular macromolecule metabolic process
28	Queen	NTA	NTA	NTA	cellular protein metabolic process
29	Queen	NTA	NTA	NTA	amino acid transmembrane transport
29	Queen	NTA	NTA	NTA	amino acid transport
29	Queen	NTA	NTA	NTA	mitotic chromosome condensation
29	Queen	NTA	NTA	NTA	chromosome condensation
29	Queen	NTA	NTA	NTA	anion transmembrane transport
30	Queen	NTA	NTA	NTA	mitotic DNA damage checkpoint
30	Queen	NTA	NTA	NTA	mitotic DNA integrity checkpoint
30	Queen	NTA	NTA	NTA	regulation of protein ubiquitination
30	Queen	NTA	NTA	NTA	negative regulation of protein ubiquitination
30	Queen	NTA	NTA	NTA	positive regulation of protein ubiquitination
31	Queen	NTA	NTA	NTA	RNA processing
31	Queen	NTA	NTA	NTA	RNA methylation
31	Queen	NTA	NTA	NTA	RNA modification
31	Queen	NTA	NTA	NTA	cellular component organization or biogenesis
31	Queen	NTA	NTA	NTA	cellular component biogenesis
32	Worker	NTA	NTA	NTA	translation
32	Worker	NTA	NTA	NTA	cellular macromolecule biosynthetic process
32	Worker	NTA	NTA	NTA	gene expression
32	Worker	NTA	NTA	NTA	macromolecule biosynthetic process
32	Worker	NTA	NTA	NTA	biosynthetic process
33	Worker	NTA	NTA	NTA	hydrogen transport
33	Worker	NTA	NTA	NTA	proton transport
33	Worker	NTA	NTA	NTA	hydrogen ion transmembrane transport
33	Worker	NTA	NTA	NTA	monovalent inorganic cation transport
33	Worker	NTA	NTA	NTA	inorganic cation transmembrane transport
34	Queen	NTA	NTA	NTA	response to misfolded protein
34	Queen	NTA	NTA	NTA	proteasome localization
34	Queen	NTA	NTA	NTA	response to topologically incorrect protein
34	Queen	NTA	NTA	NTA	cellular response to topologically incorrect protein
34	Queen	NTA	NTA	NTA	mitochondrial fusion
35	NTA	NTA	NTA	NTA	phospholipid transport
35	NTA	NTA	NTA	NTA	organophosphate ester transport
35	NTA	NTA	NTA	NTA	lipid transport
35	NTA	NTA	NTA	NTA	lipid localization
35	NTA	NTA	NTA	NTA	Kupffer's vesicle development
36	Worker	NTA	Single	NTA	synapsis
36	Worker	NTA	Single	NTA	synaptonemal complex assembly
36	Worker	NTA	Single	NTA	reciprocal meiotic recombination
36	Worker	NTA	Single	NTA	reciprocal DNA recombination
36	Worker	NTA	Single	NTA	chromosome organization involved in meiosis

No enriched GO terms could be found for module 3. *NTA* non-trait associated

### Modules are co-opted for diverse phenotypic traits

Because genes and regulatory modules involved in queen and worker phenotypes may contribute to other life-history traits, we were interested to know whether modules associated with caste explain other important phenotypic traits of social insects, including the extent of worker sterility, the number of colony queens, and invasiveness. We found eight modules that were correlated with multiple biological traits (Fig. 2; Additional file 4). Interestingly, several modules were associated with similar traits, suggesting that the evolution of some traits may be linked. e.g., modules 2, 7, and 15 were all associated with the queen caste, worker sterility, and non-invasiveness. Similarly, modules 18, 20, and 36 were associated with caste traits and single-queen colonies. More generally, it appears that the same modules play a role in influencing biological traits beyond caste differentiation. Although we focused on traits most likely arising from queen–worker differences, which was the major axis of variation in the data set, it is possible that the same modules may play a role in a wide variety of other traits.

### Module association with caste does not directly influence protein sequence evolution

**Fig. 3:**
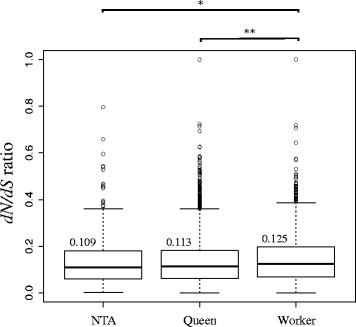
Box plots showing the distribution of *d*_N_/*d*_S_ ratios before accounting for OGG connectivity and expression levels for OGGs in non-caste-associated modules (*NTA*), OGGs in queen-associated modules (*Queen*) and OGGs in worker-associated modules (*Worker*), and calculated using PAML. The median *d*_N_/*d*_S_ values are indicated above the boxplot. OGGs in worker-associated modules had significantly higher *d*_N_/*d*_S_ than OGGs in queen-associated modules, and OGGs in non-caste-associated modules. * *p* < 0.05, ** *p* < 0.01

We ran three separate analyses to understand the effects of several explanatory variables on the *d*_N_/*d*_S._ First of all, we compared *d*_N_/*d*_S_, expression levels, and connectivity values between OGGs in caste-associated and non-caste-associated modules. OGGs in worker-associated modules had significantly higher *d*_N_/*d*_S_ than OGGs in queen-associated modules (generalized linear model (GLM), *p* = 0.01; Fig. 3), and OGGs in non-caste-associated modules (GLM, *p* = 0.016). OGGs in queen-associated modules and in non-caste-associated modules were not different to each other in terms of *d*_N_/*d*_S_ (GLM, *p* = 0.43; Fig. 3). Additionally, worker-associated genes had significantly lower connectivity than queen- (GLM, p = 0.034) and non-caste-associated genes (GLM, *p* = 0.014; Additional file 9). No significant difference in connectivity could be found between queen- and non-caste-associated genes (GLM, *p* = 0.07; Additional file 9). No significant difference in connectivity could be found between queen- and non-caste-associated genes (GLM, *p* = 0.07; Additional file 9). Furthermore, worker-associated genes had higher expression levels than queen- (GLM, *p* < 0.001) and non-caste-associated genes (GLM, *p* < 0.001; Additional file 10). No difference in expression levels could be found between queen- and non-caste-associated genes (GLM, *p* = 0.262; Additional file 10).

Second, we tested the effects of OGG connectivity and expression levels on the *d*_N_/*d*_S_ values. We found that OGG expression levels and connectivity were negatively correlated with *d*_N_/*d*_S_ (GLM, *p* < 0.01; Additional file 11), a pattern observed also in model species (e.g., yeast, [30, 32, 48]) and that may be universal to all living organisms.

Consequently, we included connectivity and expression level terms and their interactions as predictors in the GLM analysis of evolutionary rate. When taking these predictors into consideration, OGGs in queen- and worker-associated modules evolved at a higher rate than OGGs in non-caste-associated modules (GLM, *p*_*worker*_ < 0.01 and *p*_*queen*_ < 0.001), and we found no more significant differences in *d*_N_/*d*_S_ between queen- and worker-associated modules (GLM, *p* = 0.17) (Additional file 12). Instead, *d*_N_/*d*_S_ differences were best explained not by the main effects, but by the interactions between caste association, expression or connectivity. Queen-associated OGGs evolved more slowly than worker-associated OGGs with a corresponding level of connectivity. By contrast, queen-associated OGGs evolved faster than worker-associated OGGs with a similar level of expression (Additional file 12). These data suggest that caste-biased selection acts on the genome in a complex way that is modulated by expression and the regulatory interactions between genes.

### Only one gene was consistently caste-biased

**Fig. 4:**
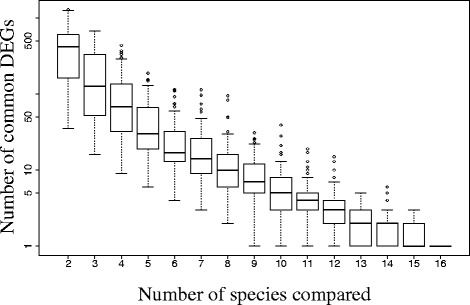
Only a single gene is consistently differentially expressed between queens and workers. The plot shows the number of caste differentially expressed genes (*DEGs*) in common in a variable number of randomly selected species (bootstrap resampling 100 times). This pairwise analysis shows either that few genes are consistently caste-biased across species or that comparison of differentially expressed genes lacks power to detect these biases. By contrast, network analysis manifested significant underlying regulatory structure, suggesting that it is a more powerful approach (Fig. 2). A similar analysis was conducted at the level of GO terms (Fig. 5)

**Fig. 5:**
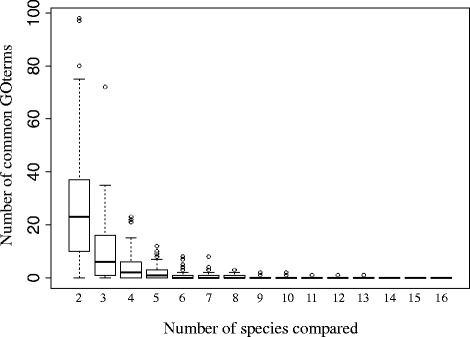
No overlap was found in the number of enriched GO terms for caste-biased genes across all 16 species. The plot shows the number of enriched GO terms for caste-biased genes in common in a variable number of randomly selected species (bootstrap resampling 100 times). The results of this analysis parallel findings at the level of individual differentially expressed genes (Fig. 4)

Although hundreds (e.g., *Formica aquilonia*) or even thousands (e.g., *Linepithema humile*) of genes showed caste-specific bias in individual species (Additional file 1), there was very little overlap among these species-specific sets (Fig. 4). In fact, when all 16 species were considered, only a single gene was differentially expressed between queens and workers (overexpressed in workers) in all species; this was the myosin light chain (Fig. 4). The myosin light chain gene is most likely a housekeeping gene that has caste-biased expression patterns owing to different demands on muscular activity by queens and workers, but it is expressed in every cell and is not a known caste-specific gene. Additionally, the worker-biased pattern of the myosin light chain may be due to a higher concentration of muscle cells present in workers compared with queens. Also, we acknowledge that a number of potential factors are likely to affect our ability to detect common caste-biased genes across our studied species (e.g., number of replicates, sampling period, statistical power over multiple datasets) that are beyond this comparative study. Additionally, at the level of functional composition, we found no GO terms consistently enriched for caste-biased genes across all 16 species (Fig. 5).

### External validation of module preservation

To verify that modules identified in this study represent technically reproducible and evolutionarily useful features, we assessed the extent of module preservation between our work and a recent WGCNA investigation that used RNA-seq to examine age-based, behavioral division of labor in workers of the pharaoh ant (*Monomorium pharaonis*) [33]. Although the earlier study did not include queens, and had a much smaller sample size (*n* = 24), 11 of the modules identified in the present study significantly overlapped with modules inferred from *M. pharaonis* behavioral data (false discovery rate (FDR) corrected *p* < 0.05; Additional file 13). Interestingly, genes in queen-associated modules, as well as worker-associated modules, were also involved in *M. pharaonis* worker division of labor, further supporting the roles of modules in multiple contexts.

## Discussion

This study suggests that morphological and physiological differences between queens and workers result from the differential expression of evolutionarily conserved sets of co-expressed genes (modules). In our analysis, large fractions of all transcriptomes could be partitioned into modules. Expression of almost all modules was correlated with queen and worker phenotypes, suggesting that they may reflect conserved regulatory control mechanisms. Many other colony-level features emerge from queen–worker interactions and phenotypes. We therefore also predicted that modules associated with queen and worker phenotypic differentiation would also be correlated with species traits that evolved in parallel, such as complete worker sterility, colony queen number, and invasiveness (Fig. 1). We found this to be the case, with the expression of several modules being associated with multiple biological traits (e.g., module 2 is associated with caste, worker sterility, queen number and invasiveness; Fig. 2). It is possible that these modules regulate the expression and evolutionary maintenance of a variety of phenotypes in multiple ant species.

Several modules were associated with the same sets of traits, such as caste phenotypes, worker reproduction and invasiveness. The traits selected for this analysis span the range from social to ecological, each evolved multiple times in the species under study; certainly other traits could be included in the model. Furthermore, it is important to note that the significance of the GLM coefficients merely suggest that they are associated with variation in eigengene expression, and that they don’t provide a complete description of the sources of variation in this data set. There may be factors, not examined in this study, which explain even more variation, and more comprehensive comparative studies will be needed to determine the best predictors. Nonetheless, some biologically suggestive patterns emerge. For example, worker sterility appears to be linked to invasiveness (Fig. 2), a pattern also found in several invasive species [49], suggesting that the two traits may be somehow transcriptionally correlated. This pattern appears to hold when considering the top invasive ant species selected by IUCN [50], and not present in our data set: *Pheidole megacephala* [51] and *Wasmannia auropunctata* [52] workers don’t have ovaries, whereas *Anoplolepis gracilipes* workers have ovaries [53], but don’t appear to reproduce (similarly to *Lasius neglectus*) [54]. This raises the intriguing possibility that the evolution of some traits may be linked in unexpected ways though sharing the same regulatory machinery, and that selection for one trait may have the effect of facilitating the evolution of other traits. These results are clearly preliminary, and worker sterility is neither a necessary nor sufficient condition for invasiveness, but we predict that some distantly related invasive ants may have similarities in gene expression profiles, and possibly even convergent patterns of selection. In the future, more work will need to assess the generality of this pattern.

In our large-scale comparative analysis we found that genes in caste-associated modules evolved faster than genes in non-caste-associated modules. Morph-biased genes are predicted to evolve more rapidly due to reduced antagonistic pleiotropy [55, 56]. In accordance with these predictions, previous studies focusing on single species have shown that genes with caste-biased expression are more likely to evolve faster than genes with unbiased expression [28, 29]. Our study is the first to examine patterns of selection across multiple species of ants with a common origin of caste differentiation. However, detecting selection is subject to a large number of factors, such as a gene’s transcriptional abundance, and its importance within the protein interaction network [57–59]. For example, gene significance (connectivity) was strongly associated with higher expression level and lower evolutionary rate in our study (GLM, *p* < 0.001), as well as in studies of other systems [34]. Expression and network effects of highly abundant genes are also well known to affect evolutionary rates, potentially confounding analysis of selection pressure [32]. Indeed, when analyzed as sole factors, caste had an effect on protein evolutionary rates, which disappeared when network effects were considered (Additional file 12). Our finding that modules correlated with caste also act in other phenotypic contexts suggests that most caste-biased genes play multiple roles. Together with recent findings suggesting that genes may fluctuate in caste-bias across development [19, 60, 61], this may explain why there is no persistent queen or worker-specific selection, i.e., caste-biased genes in one context may show other expression patterns in different developmental stages, or tissues. That being said, evolutionary rates of caste-biased genes had significant interactions with expression and connectivity (Additional file 12), suggesting that caste-biased selection may be modulated by gene expression levels and the shape of gene co-expression (or protein interaction) networks. Our results on caste bias parallel those of a recent study that showed that genes involved in another social context, nursing and foraging in an ant, differ in expression level and connectivity, and interactions with these terms are important predictors for evolutionary rates [33].

Our analysis covers only genes with orthologs in most species. We took this approach for several reasons. First, most species in our study have no available genomic resources, and we had to construct de novo transcriptomes for each of them, a technique that enables functional genomic studies, but has the potential for wide-ranging mis-assemblies [43]. By focusing on previously computed and curated orthologous gene groups [62], we were able to select the best fitting models for comparative analysis among hundreds of thousands of candidate transcripts. Second, we focused on the presence of conserved gene regulatory machinery across multiple ant species, and not on the presence of taxonomically restricted genes. The latter are likely to be involved in species-specific functions [63], but were beyond the scope of this study. Nevertheless, it is worth noting that recent studies have highlighted potential roles of novel genes in caste evolution [25, 29, 34, 64–66]. In any case, taxonomically restricted genes are likely to interact with existing regulatory pathways, and the manner in which they integrate into the conserved modules will be a fascinating topic for future research. We expect that novel genes will be poorly connected at first, which may allow greater rates of evolution [34], based on network properties alone.

A key question addressed in this study is “how does selection act on a gene important to queen versus worker phenotypes?” To answer this question we attempted to characterize gene expression by measuring their expression in whole adults. We believe that this approach approximates the proportional contribution of each gene to fitness in each caste. For example, queens have proportionally larger ovaries, and we expect higher levels of ovary-expressed genes in queen bodies overall as a result, reflecting the relative importance of ovaries to the queen phenotype. However, this approach has a number of significant limitations by greatly over-simplifying the nature of transcriptional regulation in an organism. Tissue-specific expression studies, particularly taken over the course of development, may have greater power to detect caste-specific differences, both in terms of the number of differentially expressed genes, and co-expression network structure. As a result, our data most likely represent an underestimate of the true number of modules. However, a comparative study of tissues-specific transcription at such a phylogenetic scale requires confident assignment of orthology and function, which would be difficult given the marked differences between queens and workers in the presence and size of many glands [67] and some organs (e.g., ovaries, which are absent in the workers of several lineages; Fig. 1). Future studies should focus on the comparative analysis of expression patterns in specific tissues; such studies will likely provide valuable functional insight into the function and evolution of these organs.

To date, a large number of studies, conducted in diverse systems, have found a relatively small set of genes consistently associated with caste differentiation [19, 24, 25, 36]. A recent study comparing differentially expressed caste-biased genes in an ant and a wasp likewise found no overlap in differentially expressed genes, but found some overlap in GO terms [24]. In our data set we found no enriched GO terms that were common to all 16 species (Fig. 5). However, the type II error increases multiplicatively when multiple data sets are compared, making it statistically unlikely to find elements, be it genes or GO terms, common to all of these 16 species. For instance, only a single gene was found to be differentially expressed in all species, a result that strikes us as biologically unlikely. However, this result parallels findings in honey bees (*Apis*), where microarray experiments found little overlap in differentially expressed genes within the same genus [68]. By analyzing all the genes at once, gene network analysis circumvents the problem of multiple comparisons, though at the cost of species-level resolution.

In addition WGCNA provides a more complex approach that captures system-level properties [40]. Most phenotypes involve interactions of proteins from diverse biochemical pathways. Although modules inferred from WGNCA do not necessarily correspond to biochemical pathways, or other classical components of cellular organization, WGCNA performs well in reconstructing the overall complex structure of protein–protein interaction networks [46]. In the past, for example, WGCNA has succeeded in identifying candidate genes involved in Alzheimer disease by comparing human and mouse brain transcriptomes [69], or in visualizing network structure and inferring phenotype–genotype interactions of numerous diseases such as autism [70]. Moreover, WGCNA has been used to uncover module conservation among species, and to identify crucial drivers of evolutionary changes between humans and chimpanzees [38]. Applying this approach to evolution of social insect phenotypes, we show that WGCNA recovers key modules responsible for a range of phenotypic traits, at the individual and colony levels. However, the genetic toolkit hypothesis postulates that conserved developmental pathways may have been co-opted in the evolution of reproductive division of labor, a hypothesis that has received considerable attention [20, 21, 35–37]. Although our data come from adults only, and do not allow us to test hypotheses regarding the causes of caste determination during development, or hypotheses regarding whether the same genes would be involved in multiple origins of eusociality, they do suggest that there are indeed conserved regulatory modules that are repeatedly co-opted by evolution. It will be interesting to apply network analyses to study evolution of eusociality including other ants, particularly poneroids, which were not sampled in the current study, to see how conserved the patterns are across all ants, and also across its many different origins in hymenopterans. Comparisons across origins of eusociality would reveal whether ‘toolkits’ associated with the evolution of social behavior exist. In particular, analysis of transcriptional networks during development will allow for a powerful test of the genetic toolkit hypothesis.

## Conclusions

This study is the first to investigate in-depth a single eusocial clade to understand the extent to which caste-associated regulatory architecture is preserved across taxa. We have identified a number of functionally important transcriptional modules strongly associated with caste phenotypic differences. These modules may also be co-opted for other types of phenotypic novelty, including social and ecological traits. If so, they may serve as building blocks of phenotypic innovation.

## Methods

### De novo transcriptome assembly and mapping

Detailed descriptions of sample collection, RNA extractions and cDNA synthesis can be found in Additional file 14. Workers were collected at the surface of the colony mound, and are most likely all foragers. The queens were all collected from large mature colonies. In total, we sequenced 100 libraries from whole-body queen and worker samples, representing biological replicates of each caste. The number of replicates can be found in Additional file 1. The quality of raw reads was assessed with FastQC tools (http://www.bioinformatics.bbsrc.ac.uk/projects/fastqc), and adaptor sequences were removed using cutadapt [71]. Reads were trimmed to remove low quality bases, using a dynamic trimming perl script included in the SolexaQA package [72]. In the absence of a reference genome for most species, we used a de novo assembly method to construct reference transcriptomes for each species separately for use in read mapping for caste expression profiling, using Trinity software (release 2013-02-25, default settings) [41, 42]. To remove contigs potentially from non-ant origin [73] and to include only transcripts with homology to known hymenopteran genes, only contigs which showed significant BLAST hits to the nine published hymenopteran genomes (seven ant species, *Apis mellifera*, and *Nasonia vitripennis*) were kept for further analysis (BLASTx, e-value cutoff ≤1 × 10^–5^, query coverage ≥70 %). Because most transcripts were filtered downstream during orthology assignment, we used a relatively permissive BLAST cutoff at this step.

### Quality control

Visual inspection of the multidimensional scaling (MDS) plot revealed that library replicates were similar to each other, and samples clustered more within each species than by caste (Additional file 15). RNA-seq involves a number of steps during library preparation, which may result in biases (such as batch effect). We used RNA spike-ins to evaluate the success of our RNA library preparation and sequencing. After mapping, we found a strong positive correlation between the observed ERCC spike-in expression levels and the expected abundance for each species (Additional file 16).

### Protein coding prediction and orthology assignment

The output of the Trinity pipeline is a set of transcripts, including alternatively spliced isoforms determined during graph reconstruction in the Butterfly step. These transcripts are grouped into gene components, which represent likely multiple isoforms. To minimize effects of possible isoform variation between species, we kept only the longest component for further analysis. Next, we used two software packages, OrfPredictor [74] and FrameDP [75], to predict protein coding sequences of filtered contigs. We used OGGs for the sub-family Formicidae from OrthoDB7 as the reference for our own orthology assignment. Protein sequences were downloaded from the OrthoDB FTP site [62], and aligned using PRANK (v. 140603) [76], using a neighbor joining guide tree generated by MAFFT (v. 7.164) [77]. These alignments were used to generate hidden Markov models (HMMs) using HMMER (v. 3.1b1; http://hmmer.org). We then used these models within the HaMStr [78] pipeline to assign each gene prediction to an OrthoDB OGG, using default settings. Only OGGs with contigs from at least four species were retained for further analysis. This method selected the best fit among alternative open reading frame predictions, and assigned genes to independently established OGGs, which should facilitate future comparative work.

### Caste-biased gene expression

Paired-end reads were mapped to the de novo transcriptomes using RSEM [79], and the resulting expected counts were used in the subsequent differential gene expression analysis with the R Bioconductor package EdgeR [80]. Reads generated by the three queen and the three worker samples were used as replicates. Transcripts without, or with very low, read counts were filtered out before performing the test, using the threshold determined by the detection limit of the RNA spike-in analysis (Additional file 1). As recommended by EdgeR, TMM normalization was applied to account for compositional difference between libraries, and expression differences were considered significant at a FDR < 0.05. Pairwise comparison analyses between castes were performed for all species separately. Subsequently, differentially expressed genes were assigned to their respective OGGs, and caste-bias expressions were compared within each OGG to find common caste differentially expressed genes among all species.

In order to verify whether caste-biased gene expression has a phylogenetic signal and also that our phylogenetic sampling was not affecting our ability to detect commonly differentially expressed genes, we compared the number of commonly differentially expressed genes across seven *Formica* species and across seven randomly selected non-*Formica* species. We found a very similar trend for both pairwise relationships, and very few genes were found commonly differentially expressed in both cases (non-*Formica* species, 7; *Formica* species, 21; Additional file 17).

### Generation of weighted gene co-expression networks and identification of functional modules

Trimmed mean of M-values normalization was applied to the raw count expression data (WGCNA) using the R package EdgeR [80]. Subsequent weighted gene co-expression network analysis was conducted using the R package WGCNA [40]. The input dataset consisted of a matrix with 100 columns, each corresponding to a queen or worker RNA-seq library from the 16 species, and 9859 lines, each representing one OGG expression level. If multiple transcripts from the same species were present in one OGG, their expression levels were averaged. This data set was first filtered to remove OGGs (lines) with too many missing values, following WGCNA cutoff threshold recommendations (Additional file 3). Additionally, one outlier sample (column) was filtered out following the WGCNA package guidelines, and consequently removed from the differential gene expression analysis described above (Additional file 18). A soft thresholding power of 8 was chosen based on the criterion of approximate scale-free topology (Additional file 19). After calculating topological overlap values for all pairs of orthologous gene groups, a hierarchical clustering algorithm identifies modules of highly interconnected genes. Subsequently, modules of highly co-expressed OGGs were merged together using a cutoff value of 0.2 and the minimum module size was set to 30 (Additional file 20).

### Evolutionary rate analysis

Because our transcriptomes may contain stochastic variation in the number of reconstructed paralogs, for analysis of evolutionary rate only a single gene prediction per species, the one most closely matching the reference HMM, was chosen per OrthoDB gene using HaMStr. These genes were re-aligned using PRANK [76] as protein sequences. Confidence of these alignments was assessed using GUIDANCE [81], PRANK realignments based on 32 bootstrap replicates, using the heads-or-tails method. Residues with GUIDANCE confidence less than 0.9 were replaced by Ns. Genes with fewer than 150 non-ambiguous nucleotides were eliminated from the analysis. The best maximum likelihood tree was computed with codonPhyML (v. 1.00) [82]. The Codeml module from PAML (v. 4.4), [83] was used to estimate *d*_N_/*d*_S_ of different genes using these trees and alignments, with branch lengths and transition/transversion estimates from codonPhyML as starting values. We estimated overall *d*_N_/*d*_S_ for each OGG using a one-ratio model (model = 0), providing a single estimate for each OGG to match other single OGG metrics, such as connectivity and expression.

Three separate GLM analyses were conducted using the glm function in R with 1000 bootstrap pseudoreplicates. First, we investigated the effects of caste on *d*_N_/*d*_S_, using OGG *d*_N_/*d*_S_ values as the main effects and caste OGG association as the explanatory variables, GLM = *d*_N_/*d*_S_ ~ Caste. A similar procedure was used with OGG connectivity and expression levels, GLM = Connectivity ~ Caste and GLM = Expression ~ Caste.

Second, we investigated the effect of OGG connectivity and expression levels (explanatory variables) on the *d*_N_/*d*_S_ values (main effect), GLM = *d*_N_/*d*_S_ ~ Exp + Connectivity.

Third, we used a single model to investigate the combined effects of OGG connectivity, OGG expression levels and biological traits (explanatory variables) on OGG *d*_N_/*d*_S_ values (main effect) GLM = *d*_N_/*d*_S_ ~ Connectivity * Caste + Expression * Caste.

For all three GLM analyses, biological trait effects were derived from module association with queen, worker or NTA (Fig. 2), and *d*_N_/*d*_S_, connectivity and expression level values were exponentially transformed to reach a normal distribution before being processed. Detailed scripts can be found in https://github.com/MikheyevLab/Comparative-transcriptomics-of-ants under a MIT license.

### Functional annotation of co-expressed modules

GO terms for all genes were determined using Blast2GO (using BLASTp with an e-value cutoff ≤ 10^–3^) [84]. We used the GOstats package for R [85] to conduct GO term enrichment analysis on gene sets included in the modules described above, using the set of all genes for which GO terms were available as the universe.

### Module preservation

We also conducted module preservation statistics using WGCNA modules retrieved from a recent study of worker behavior [33, 86]. We compared the extent of module preservation in an independent data set by checking whether there was correspondence in module assignment between this study and an earlier study of behavioral polyethism in *M. pharaonis*, which also used WGCNA [33]. Orthologs of *M. pharaonis* genes were selected using BLAST. We then calculated how often genes were classified as belonging to the same module by both studies [86]. Statistical significance was determined using Fisher's exact test, adjusted for multiple comparisons using FDR with the FDR set at 0.05.

### Phylogenetic tree construction

We used OGG alignments produced for the PAML analysis that had no missing data to construct a phylogenetic tree of species relationships using RAxML (v. 8) [87]. The data set contained 1427 genes and 3.59 Mb of sequence, and the analysis was partitioned by gene and conducted under a GTRGAMMAI model.

### Module and phenotype relationship

In order to determine the relationship between modules and phenotypic traits (e.g., caste, worker sterility, queen number, invasiveness), we calculated the average signed normalized gene expression values called an “eigengene”. The eigengene is defined as the first principal component of a module and represents the gene expression profiles. One eigengene value per sample and per module was calculated. To investigate if eigengenes were associated with the external phenotypic traits, we applied a Markov Chain Monte Carlo method with phylogenetically correlated random effects, implemented by the software package MCMCglmm [45], which was run in R 3.3.1 [88]. We first calculated the inverse of the matrix of phylogenetic correlation, using an ultrametric tree computed using Sanderson’s non-parametric rate smoothing method [89]. The best smoothing parameter, lambda, was chosen by cross-validation over a range of possible values [90] and was set to 0.1. We used non-informative priors corresponding to an inverse-Gamma distribution with shape and scale parameters equal to 0.01. MCMC burn-in was set to 150,000, and 500,000 simulations were carried out in total. Convergence, effective sample size and mixing were controlled for. The GLMM approach provides a convenient means of testing the correlation of multiple traits with module eigengenes by using a single model relating eigengene expression to caste phenotype and all species traits (worker sterility, queen number, and invasiveness; Input table Additional file 21).

### Availability of supporting data

The raw reads of the transcriptome are publicly available in the DNA Data Bank of Japan under bioproject ID PRJDB4088, sample accession numbers ID SAMD00035735-SAMD00035834; *Formica aquilonia* LH381539-LH513652, *Formica cinerea* LH513653-LH652103, *Formica exsecta* LH652104-LH973351, *Formica fusca* LI000001-LI121692, *Formica pratensis* LI121693-LI219804, *Formica pressilabris* LI219805-LI349988, *Formica truncurum* LI349989-LI476587, *Lasius neglectus* LI476588-LI563515, *Lasius turcicus* LI563516-LI670604, *Linepithema humile* LI670605-LI795928, *Monomorum chinense* LI795929-LI926639, *Monomorium pharaonis* LJ000001-LJ120855, *Myrmica rubra* LJ120856-LJ206166, *Myrmica ruginodis* LJ206167-LJ284088, *Myrmica sulcinodis* LJ284089-LJ356044, *Solenopsis invicta* (monogynous form) LJ356045-LJ530869, *Solenopsis invicta* (polygynous form) LJ530870-LJ707314. All transcriptome assemblies can be found on Fourmidable (http://antgenomes.org/downloads/) [91].

All source codes used for the analysis are provided at https://github.com/MikheyevLab/Comparative-transcriptomics-of-ants under a MIT license, and a detailed workflow of the WGCNA analysis is provided in Additional file 22.

### Ethics approval

No ethical approval was required.

## Additional files

Additional file 1: Table S1. Number of queens and workers used for RNA pooling before library preparation. Three replicates per species per caste were sequenced using an equal number of samples in each of them. Number of pooled *Formica exsecta* samples can be found in [19]. Number of de novo assembled contigs using Trinity before and after filtering, given with the transcriptomes’s size and number of reads. See Methods for full description of the filtering steps. Number of caste differentially expressed genes found in each species using EdgeR (Queens, number of queen upregulated genes; Workers, number of worker upregulated genes; Non DE, number of non-differentially expressed genes; Initial count, number of contigs analysed before cutoff; Cutoff, cutoff limit from RNA-spike-in analysis). The number of differentially expressed genes between queen and worker varied among species, from 323 genes (7.4 % of the total number of genes kept for the analysis) in *Formica aquilonia* to 5502 genes (72 %) in *Linepithema humile* (*S. invicta* mono, *Solenopsis invicta* monogynous form; *S. invicta* poly, *Solenopsis invicta* polygynous form). No queens were found for *Lasius turcicus*. (XLSX 42 kb)

Additional file 2: Table S2. Dataframe used as input for the WGCNA analysis. Each column represents one sample analyzed and each row represents the expression level of one OGG across all samples. (XLSX 7837 kb)

Additional file 3: Figure S1. Expression levels of orthologous gene groups removed (*blue*) and kept (*red*) for WGCNA analysis. WGCNA pre-cleaning step removed data with excessive missing values, which may impact our ability to detect co-expression patterns. On average, expression data were not available for 26 samples (out of 100) for the removed OGGs (2432 OGGs), and only 7 samples for the contigs that were kept for further analysis (7427 OGGs). (PDF 12 kb)

Additional file 4: Table S3. Results of the MCMC general linear model (GLM) with 500,000 iterations testing the influence of biological traits on module eigengenes and accounting for the effects of phylogeny. The expression of 32 modules was significantly correlated with one of the two female castes. The modules associated with caste were also found to be correlated with several important phenotypic traits (worker sterility, the number of queens per colony and invasiveness). We found that eight modules showed significant interactions with multiple traits, suggesting that these modules play a role in biological traits beyond caste differentiation. (XLSX 3332 kb)

Additional file 5: Table S4. Number of differentially expressed genes (*DEGs*) present in each module (*Caste*, association of the module with either queen (*Queen*) or worker traits (*Worker*), or not associated (*NTA*); *No. Queen DEGs*, number of queen upregulated genes belonging to the module; *No Worker DEGs*, number of worker upregulated genes belonging to the module; *No Non DEGs*, number of genes non-differentially expressed belonging to the module; *% DEGs*, percentage of DEGs compared with the total number of genes found in the modules). (PDF 30 kb)

Additional file 6: Figure S2. Visualization of two caste-associated modules (*Queen* and *Worker*). The graph represents genes (nodes) connected by edges showing correlation in gene expression. Central genes (hub genes) which have multiple connections to other genes and their biological functions are indicated. Hub genes have high probabilities of being essential for biological functions [92]. (PDF 252 kb)

Additional file 7: Table S5. List of blast annotations for each OGG using BLASTp. (XLSX 243 kb)

Additional file 8: Table S6. List of enriched GO term for each module. The GOstats package for R [85] was used to conduct GO term enrichment analysis. No enriched GO term could be found for module 3. (XLSX 293 kb)

Additional file 9: Figure S3. Box plots showing the distribution of connectivity rates for OGGs in non-caste-associated modules (NTA), OGGs in queen-associated modules (Queen) and OGGs in worker-associated modules (Worker), and calculated using WGCNA. The median connectivity values are indicated above the boxplot. OGGs in worker-associated modules had significantly lower connectivity rates than OGGs in queen-associated modules (GLM, p = 0.034) and in non-caste-associated modules (GLM, *p* = 0.014) * *p* < 0.05.. (PDF 50 kb)

Additional file 10: Figure S4. Box plots showing the distribution of expression levels for OGGs in non-caste-associated modules (*NTA*), OGGs in queen-associated modules (*Queen*) and OGGs in worker-associated modules (*Worker*), and calculated using RSEM. The median *expression* values are indicated above the boxplot. OGGs in worker-associated modules had significantly higher expression values than OGGs in queen-associated modules (GLM, *p* < 0.001) and in non-caste-associated modules (GLM, *p* < 0.001) *** *p* < 0.001. (PDF 59 kb)

Additional file 11: Table S7. Effects of OGG expression levels and connectivity on *d*_N_/*d*_S_. OGGs expression levels and connectivity were negatively correlated with *d*_N_/*d*_S_. Consequently, we included these terms and their interactions as predictors in the GLM analysis of evolutionary rate. (XLSX 26.8 kb)

Additional file 12: Table S8. Results of the general linear model (GLM) with bootstrapping (1000 times) testing the influence of OGGs’ connectivity, expression levels and phenotypic traits on modules’ rates of evolution (*NTA* non-traits-associated modules). * *p* < 0.05, **p < 0.01, ***p < 0.001. After accounting for the effects of network topology and expression levels, *d*_N_/*d*_S_ were not different for OGGs within modules associated with any of the phenotypes we investigated. Because a single module may be involved in multiple phenotypes, its constituent genes may experience different selective pressures in different contexts. (XLSX 39 kb)

Additional file 13: Figure S5. Module preservation in an independent data set. To validate the existence of a module, it is desirable to show that it is preserved in an independent test network [86]. The matrix shows the number of genes assigned to modules by Mikheyev and Linksvayer [33] in a study of forager behavioral polyethism in *Monomorium pharaonis*, and in the present study. If the modules are truly employed in different contexts, we expected some module overlap between the two data sets, despite the fact that the Mikheyev and Linksvayer study only focused on workers. Modules with a significant overlap in genes (FDR adjusted Fisher's exact test *p* < 0.05) are highlighted in shades of *red*. Both studies use the same WGCNA software for module definition, but independent data sources. The existence of significant overlaps suggests that many modules are reproducible in a variety of contexts. (PDF 60 kb)

Additional file 14: Supplementary material and methods. (PDF 130 kb)

Additional file 15: Figure S6. Multidimensional scaling (MDS) plot showing transcriptional similarity between the samples. Samples tend to cluster more by species than by caste, and phylogenetic information is well characterized, with subfamilies forming clear clusters. (PDF 74 kb)

Additional file 16: Figure S7. Plot of the observed versus expected log2 ratio of the ERCC expression levels for each species and each library constructed. The plots showed a positive relationship and revealed that library construction was successful. (PDF 765 kb)

Additional file 17: Figure S8. The number of caste differentially expressed genes in common across all seven *Formica* species and across seven randomly selected non-*Formica* species (bootstrap resampling 100 times). This pairwise analysis shows a similar trend for both plot with very low overlap of differentially expressed genes, even despite the phylogenetic relationship across *Formica* species. (PDF 42 kb)

Additional file 18: Figure S9. WGCNA sample clustering based on gene expression patterns used to detect outliers. One replicate of *S. invicta* queen sample was removed from WGCNA and further expression analysis. (PDF 44 kb)

Additional file 19: Figure S10. Scale free topology criterion with a R^2 threshold of 0.9. A soft threshold power of 8 was chosen. (PDF 44 kb)

Additional file 20: Figure S11. Dendogram of OGG gene expression patterns and module colors. The network analysis of gene expression in ants identifies distinct modules of co-expressed genes. The dendrogram is produced by hierarchical clustering of 7427 orthologous gene groups based on topological overlap. (PDF 324 kb)

Additional file 21: Table S9. Eigengene values calculated for each sample for each module. Eigengene values summarize the expression profile of each module. These values were subsequently used to relate the modules with external information (*Caste*, *Queen number*, *Worker sterility* and *Invasiveness*). (XLSX 75.3 kb)

Additional file 22: Table S10. Detailed workflow of the WGCNA analysis. (PDF 520 kb)
