# Erratum to: Comparative transcriptomics reveals the conserved building blocks involved in parallel evolution of diverse phenotypic traits in ants

**DOI:** 10.1186/s13059-016-1048-3

**Published:** 2016-08-25

**Authors:** Claire Morandin, Mandy M. Y. Tin, Sílvia Abril, Crisanto Gómez, Luigi Pontieri, Morten Schiøtt, Liselotte Sundstrom, Kazuki Tsuji, Jes Søe Pedersen, Heikki Helantera, Alexander S. Mikheyev

**Affiliations:** 1 Centre of Excellence in Biological Interactions, Department of Biological and Environmental Sciences, University of Helsinki, Helsinki, Finland; 2 Tvärminne Zoological Station, University of Helsinki, J.A. Palménin tie 260, FI-10900 Hanko, Finland; 3 Okinawa Institute of Science and Technology, 1919-1 Tancha Onna-son, Kunigami-gun, Okinawa 904-0412 Japan; 4 Department of Environmental Sciences, University of Girona, Campus Montilivi, 17071 Girona, Spain; 5 Centre for Social Evolution, Department of Biology, University of Copenhagen, Universitetsparken 15, DK-2100 Copenhagen, Denmark; 6 Department of Subtropical Agro-Environmental Sciences, University of the Ryukyus, Senbaru 1, Nishihara, Okinawa 903-0213 Japan; 7 Research School of Biology, Australian National University, Canberra, ACT 0200 Australia

## Erratum

After the publication of this work [1] it was noticed that the mapping between column 1 and 2 in the Additional file 7 – Table S5 was incorrect.

Please see the corrected additional file below.

The publisher apologises for this error.

## Additional file

Additional file 7: Table S5. List of blast annotations for each OGG using BLASTp. (XLSX 222 kb)
