# Supplementary figures and images for: Comparative transcriptomics reveals the conserved building blocks involved in parallel evolution of diverse phenotypic traits in ants
Source: Genome Biol. 2016 Mar 7;17:43. doi: 10.1186/s13059-016-0902-7 (PMC4780134; doi:10.1186/s13059-016-0902-7)

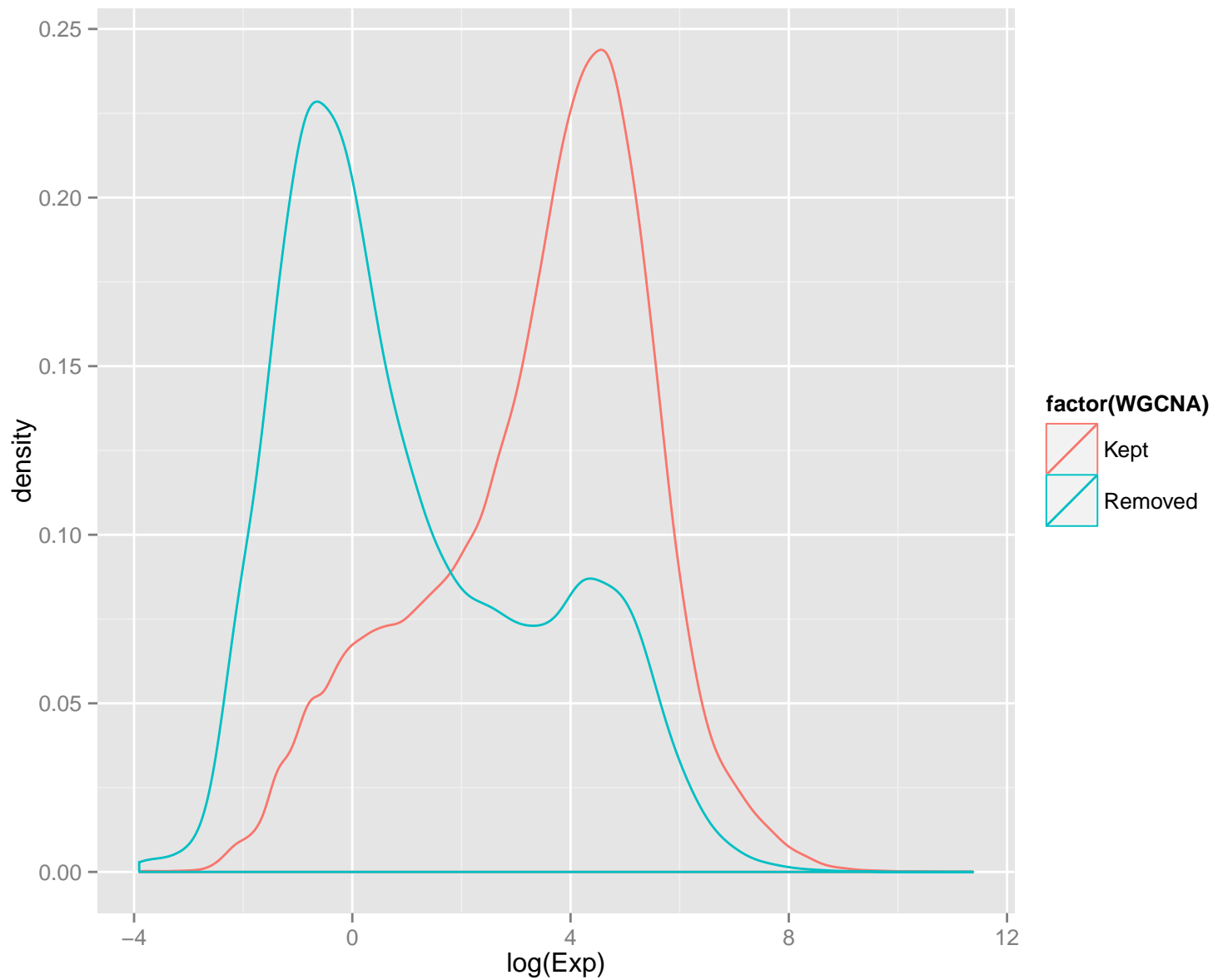

Supplement: Supplementary file 3 — Expression levels of orthologous gene groups removed (blue) and kept (red) for WGCNA analysis. WGCNA pre-cleaning step removed data with excessive missing values, which may impact our ability to detect co-expression patterns. On average, expression data were not available for 26 samples (out of 100) for the removed OGGs (2432 OGGs), and only 7 samples for the contigs that were kept for further analysis (7427 OGGs). (PDF 12 kb) [file 13059_2016_902_MOESM3_ESM.pdf]

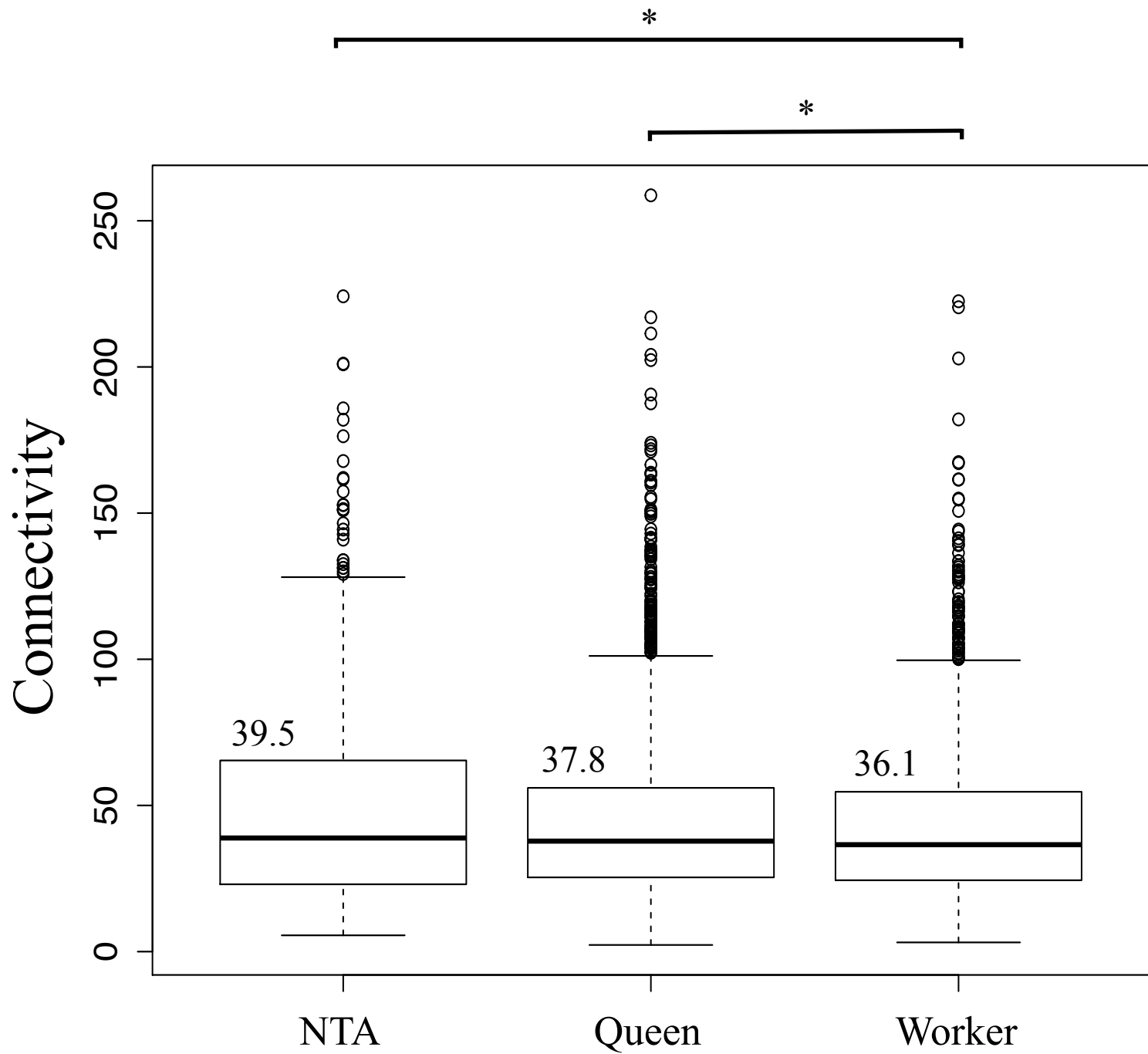

Supplement: Supplementary file 9 — Box plots showing the distribution of connectivity rates for OGGs in non-caste-associated modules (NTA), OGGs in queen-associated modules (Queen) and OGGs in worker-associated modules (Worker), and calculated using WGCNA. The median connectivity values are indicated above the boxplot. OGGs in worker-associated modules had significantly lower connectivity rates than OGGs in queen-associated modules (GLM, p = 0.034) and in non-caste-associated modules (GLM, p = 0.014) * p < 0.05.. (PDF 50 kb) [file 13059_2016_902_MOESM9_ESM.pdf]

Expression levels

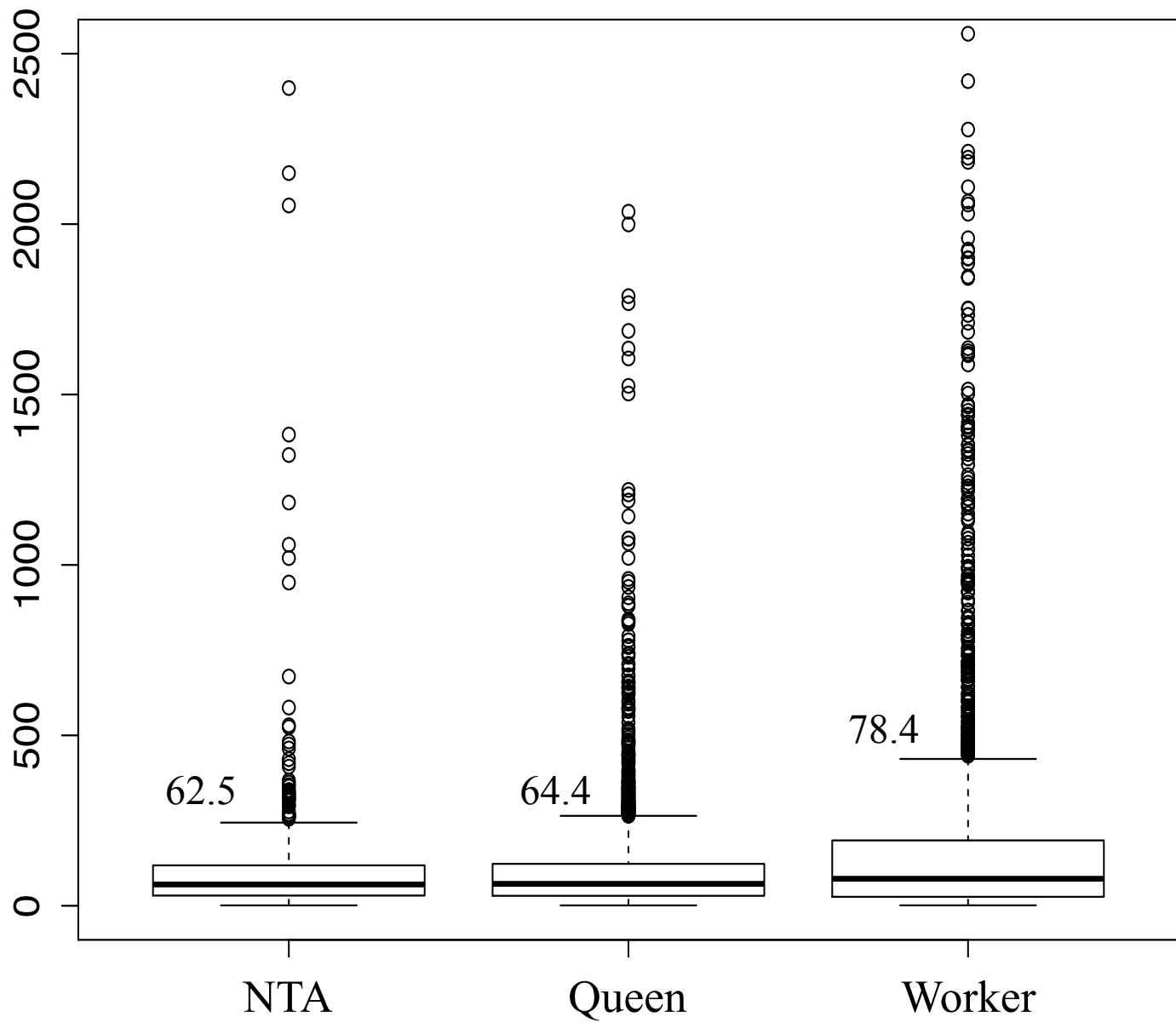

Supplement: Supplementary file 10 — Box plots showing the distribution of expression levels for OGGs in non-caste-associated modules (NTA), OGGs in queen-associated modules (Queen) and OGGs in worker-associated modules (Worker), and calculated using RSEM. The median expression values are indicated above the boxplot. OGGs in worker-associated modules had significantly higher expression values than OGGs in queen-associated modules (GLM, p < 0.001) and in non-caste-associated modules (GLM, p < 0.001) *** p < 0.001. (PDF 59 kb) [file 13059_2016_902_MOESM10_ESM.pdf]

Worker forager modules

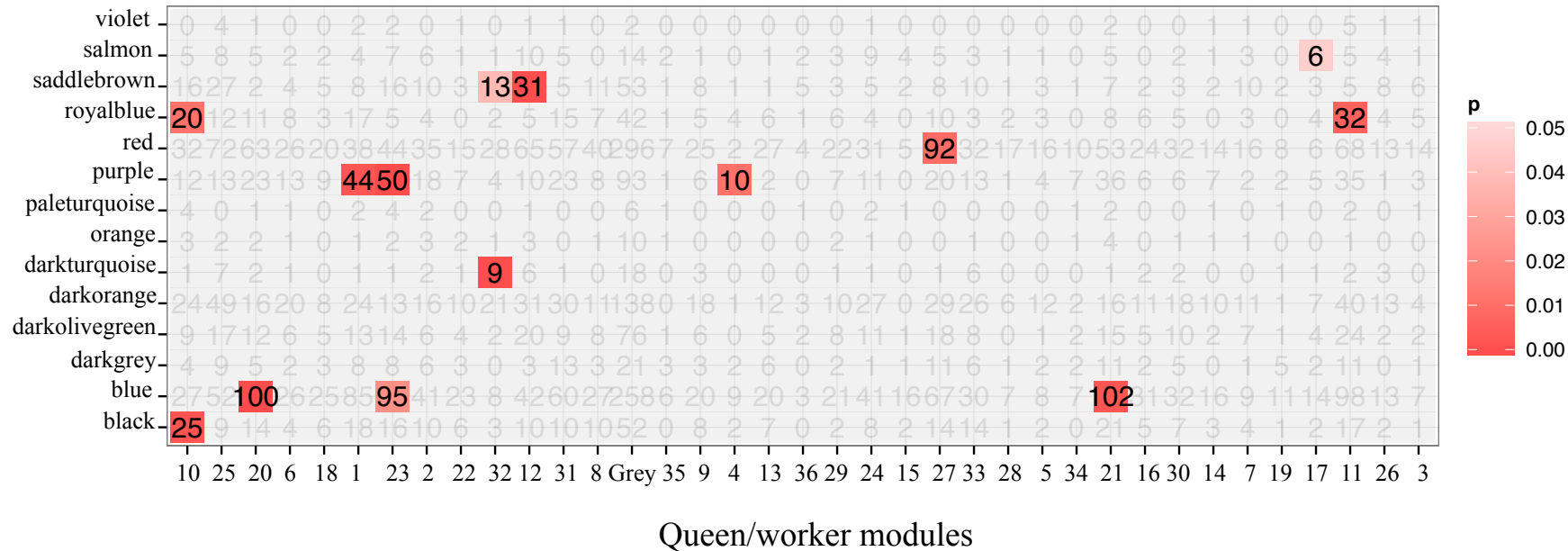

Supplement: Supplementary file 13 — Module preservation in an independent data set. To validate the existence of a module, it is desirable to show that it is preserved in an independent test network [86]. The matrix shows the number of genes assigned to modules by Mikheyev and Linksvayer [33] in a study of forager behavioral polyethism in Monomorium pharaonis, and in the present study. If the modules are truly employed in different contexts, we expected some module overlap between the two data sets, despite the fact that the Mikheyev and Linksvayer study only focused on workers. Modules with a significant overlap in genes (FDR adjusted Fisher's exact test p < 0.05) are highlighted in shades of red. Both studies use the same WGCNA software for module definition, but independent data sources. The existence of significant overlaps suggests that many modules are reproducible in a variety of contexts. (PDF 60 kb) [file 13059_2016_902_MOESM13_ESM.pdf]

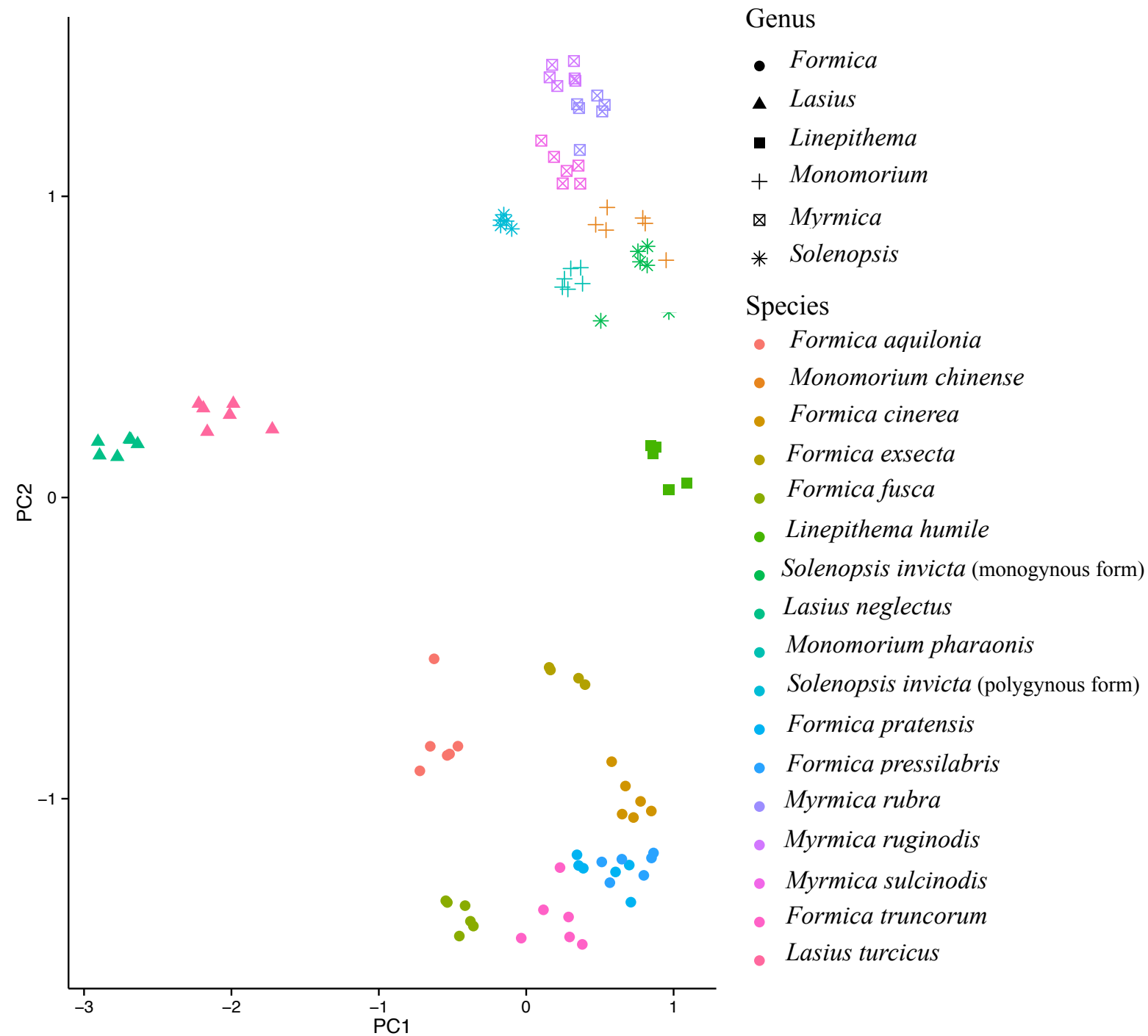

Supplement: Supplementary file 15 — Multidimensional scaling (MDS) plot showing transcriptional similarity between the samples. Samples tend to cluster more by species than by caste, and phylogenetic information is well characterized, with subfamilies forming clear clusters. (PDF 74 kb) [file 13059_2016_902_MOESM15_ESM.pdf]

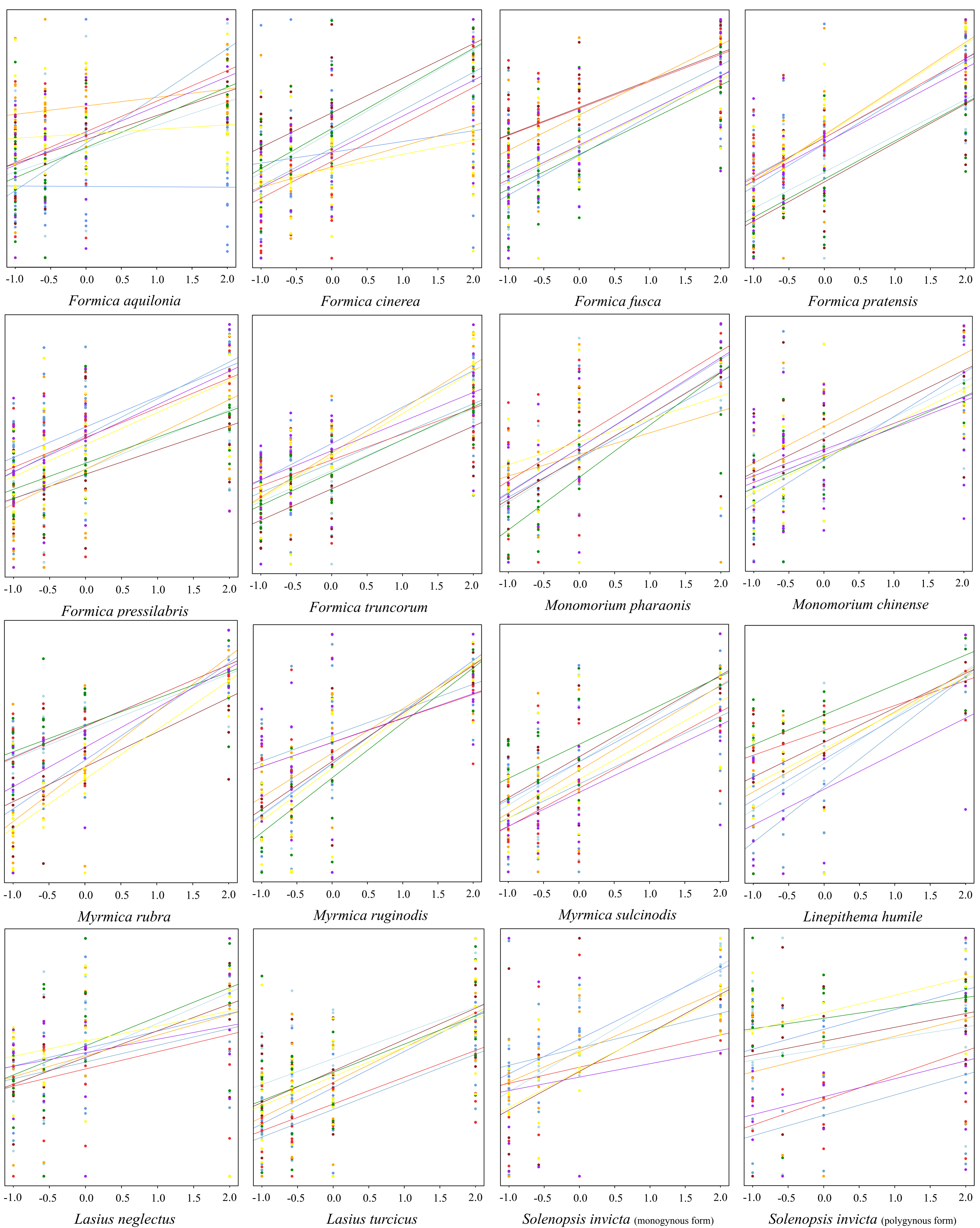

Supplement: Supplementary file 16 — Plot of the observed versus expected log2 ratio of the ERCC expression levels for each species and each library constructed. The plots showed a positive relationship and revealed that library construction was successful. (PDF 765 kb) [file 13059_2016_902_MOESM16_ESM.pdf]

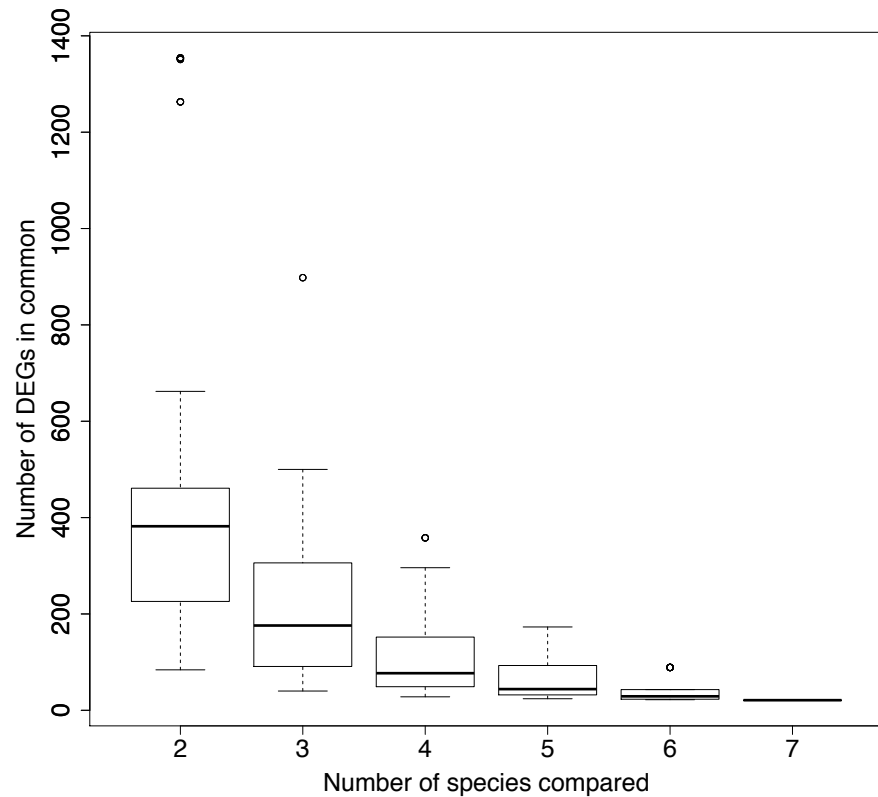

*Formica* species

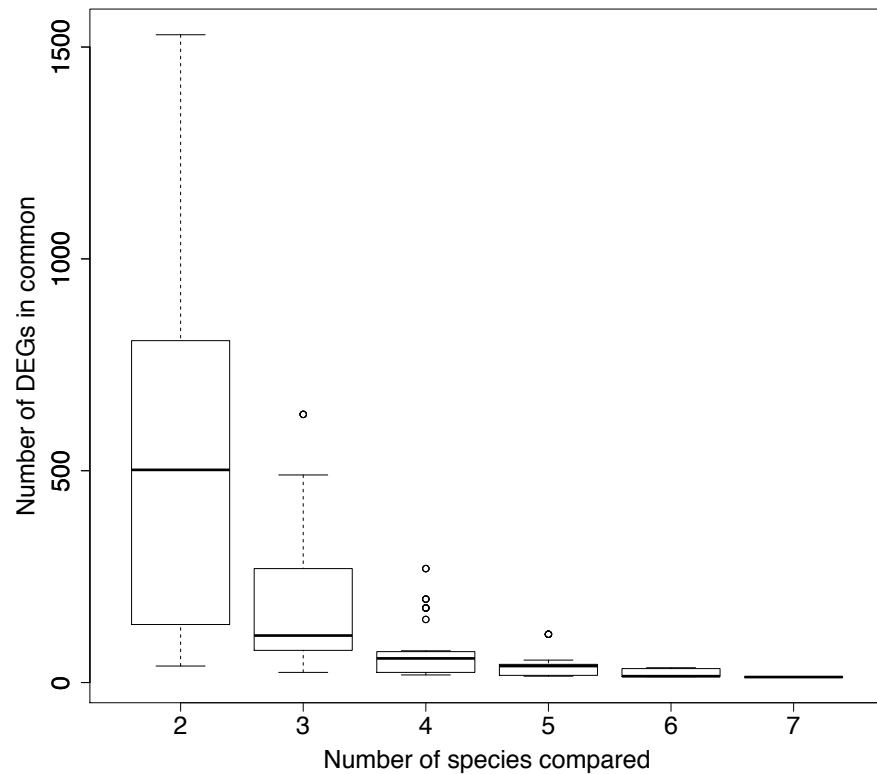

Non-*Formica* species

Supplement: Supplementary file 17 — The number of caste differentially expressed genes in common across all seven Formica species and across seven randomly selected non-Formica species (bootstrap resampling 100 times). This pairwise analysis shows a similar trend for both plot with very low overlap of differentially expressed genes, even despite the phylogenetic relationship across Formica species. (PDF 42 kb) [file 13059_2016_902_MOESM17_ESM.pdf]

Height

0 50000 100000 150000 200000 250000 300000

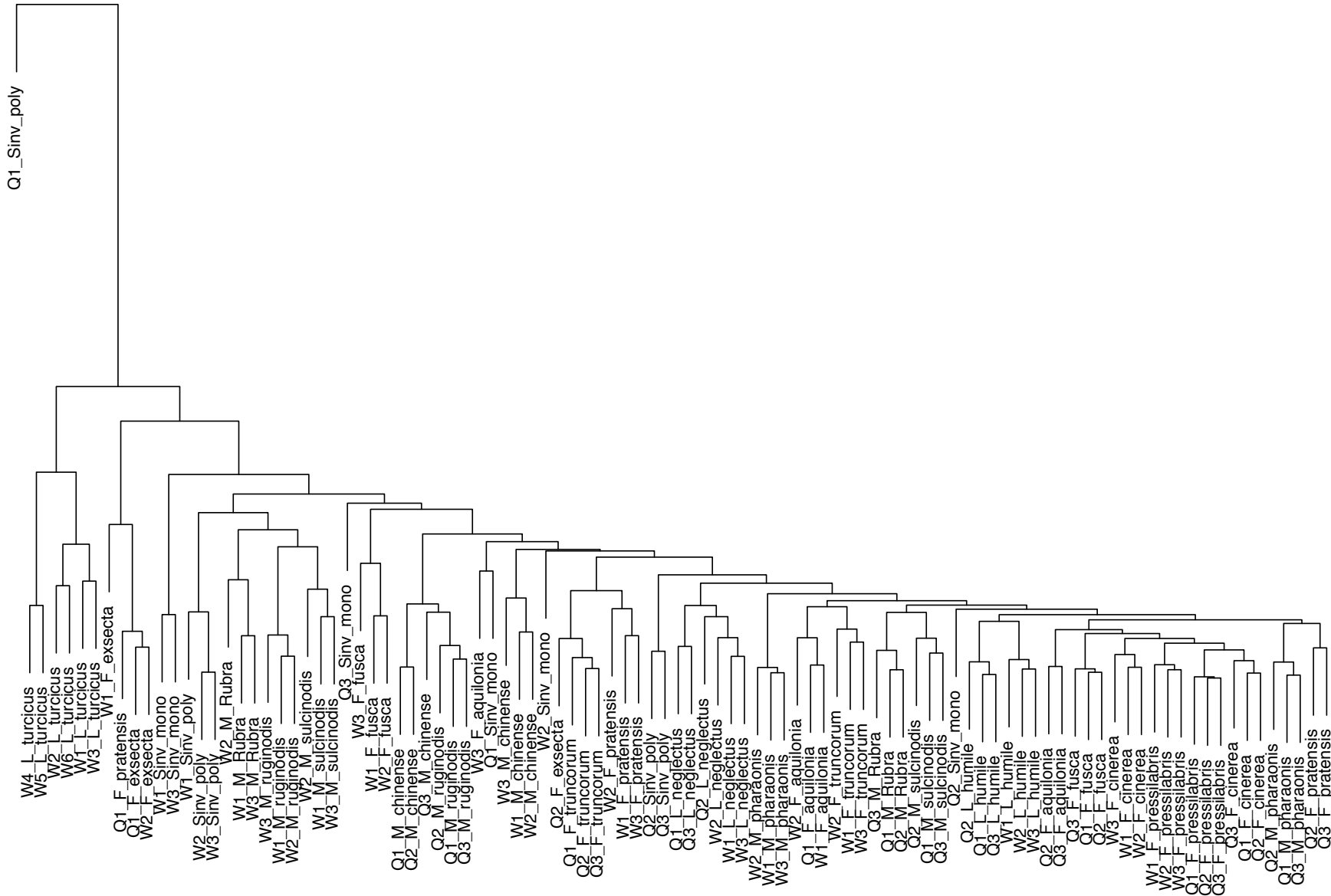

Supplement: Supplementary file 18 — WGCNA sample clustering based on gene expression patterns used to detect outliers. One replicate of S. invicta queen sample was removed from WGCNA and further expression analysis. (PDF 44 kb) [file 13059_2016_902_MOESM18_ESM.pdf]

# Cluster Dendrogram

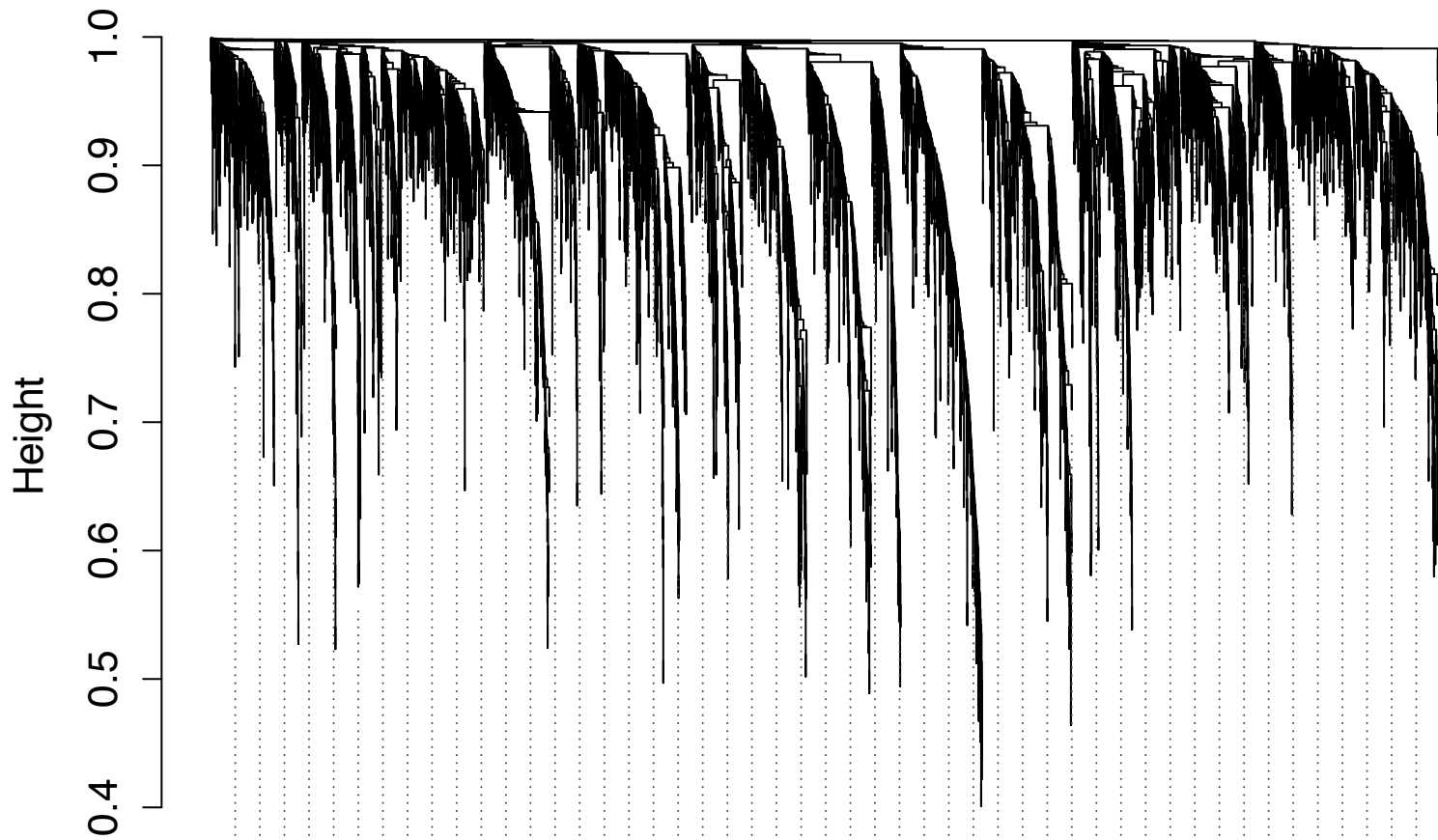

Module assignment before cut-off

Module assignment after cut-off  
(value of 0.2)

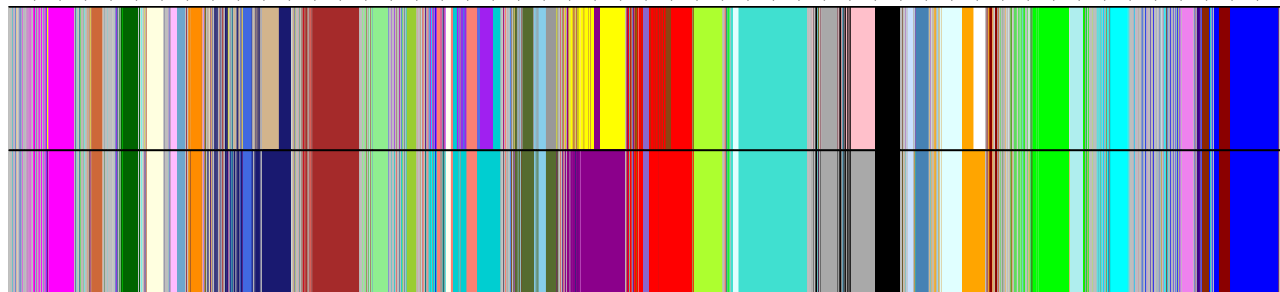

Supplement: Supplementary file 20 — Dendogram of OGG gene expression patterns and module colors. The network analysis of gene expression in ants identifies distinct modules of co-expressed genes. The dendrogram is produced by hierarchical clustering of 7427 orthologous gene groups based on topological overlap. (PDF 324 kb) [file 13059_2016_902_MOESM20_ESM.pdf]
