# Supplementary material for: Comparative transcriptomics reveals the conserved building blocks involved in parallel evolution of diverse phenotypic traits in ants
Source: Genome Biol. 2016 Mar 7;17:43. doi: 10.1186/s13059-016-0902-7 (PMC4780134; doi:10.1186/s13059-016-0902-7)
Supplement: Supplementary file 5 — Number of differentially expressed genes (DEGs) present in each module (Caste, association of the module with either queen (Queen) or worker traits (Worker), or not associated (NTA); No. Queen DEGs, number of queen upregulated genes belonging to the module; No Worker DEGs, number of worker upregulated genes belonging to the module; No Non DEGs, number of genes non-differentially expressed belonging to the module; % DEGs, percentage of DEGs compared with the total number of genes found in the modules). (PDF 30 kb) [file 13059_2016_902_MOESM5_ESM.pdf]

Module	Caste	Nº Queens	Nº Workers	Nº Non DEGs	% DEGs
1	Queen	51	158	880	23,75
2	Queen	17	455	952	49,58
3	Worker	1150	363	6395	23,66
4	Queen	75	179	1253	20,27
5	Queen	61	86	765	19,22
6	Queen	45	290	1168	28,68
7	Worker	100	74	1142	15,24
8	Worker	259	211	2353	19,97
9	Worker	131	117	1529	16,22
10	Worker	679	360	6459	16,09
11	NTA	112	18	878	14,81
12	Worker	205	58	1298	20,26
13	Queen	134	41	551	31,76
14	NTA	206	616	2543	32,32
15	Queen	1307	124	4194	34,12
16	Queen	116	474	2849	20,71
17	Worker	9	107	582	19,93
18	Queen	55	29	719	11,68
19	NTA	572	74	1412	45,75
20	Queen	214	70	1884	15,07
21	Queen	77	16	729	12,76
22	Queen	173	367	2087	25,87
23	Queen	82	492	2448	23,45
24	Worker	150	1238	4800	28,92
25	Worker	139	100	1249	19,14
26	Worker	321	146	2901	16,10
27	Queen	166	395	2742	20,46
28	Queen	1515	138	3059	54,04
29	Queen	42	38	501	15,97
30	Queen	478	194	5413	12,41
31	Queen	596	285	5107	17,25
32	Worker	217	107	1698	19,08
33	Worker	299	180	1996	24,00
34	Queen	996	153	4847	23,71
35	NTA	317	1106	4623	30,78
36	Worker	102	604	3440	20,52
