# Supplementary material for: Comparative transcriptomics reveals the conserved building blocks involved in parallel evolution of diverse phenotypic traits in ants
Source: Genome Biol. 2016 Mar 7;17:43. doi: 10.1186/s13059-016-0902-7 (PMC4780134; doi:10.1186/s13059-016-0902-7)
Supplement: Supplementary file 6 — Visualization of two caste-associated modules (Queen and Worker). The graph represents genes (nodes) connected by edges showing correlation in gene expression. Central genes (hub genes) which have multiple connections to other genes and their biological functions are indicated. Hub genes have high probabilities of being essential for biological functions [92]. (PDF 252 kb) [file 13059_2016_902_MOESM6_ESM.pdf]

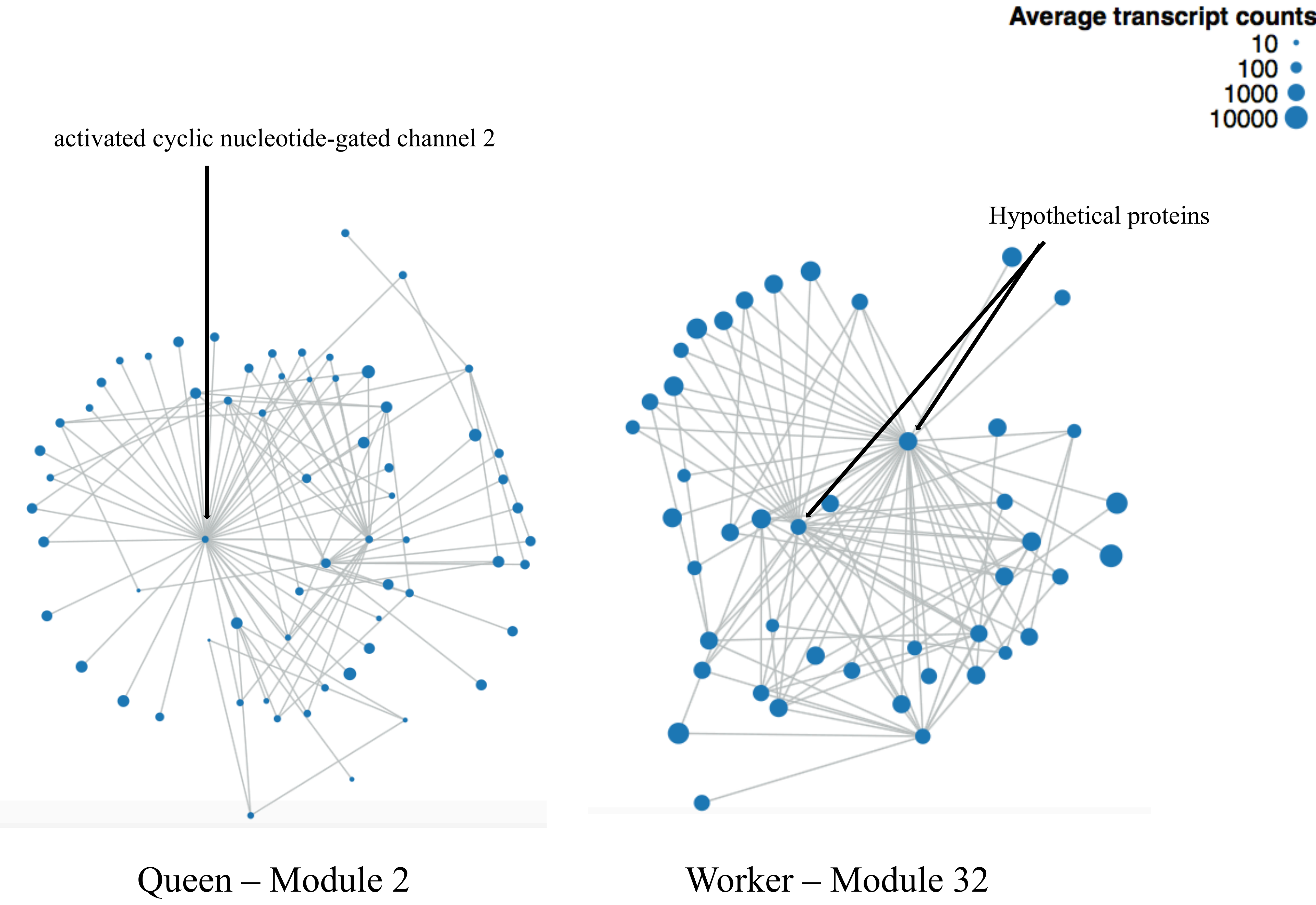
Average transcript counts
10
100
1000
10000
activated cyclic nucleotide-gated channel 2
Hypothetical proteins
Queen – Module 2
Worker – Module 32
