# Supplementary material for: Comparative transcriptomics reveals the conserved building blocks involved in parallel evolution of diverse phenotypic traits in ants
Source: Genome Biol. 2016 Mar 7;17:43. doi: 10.1186/s13059-016-0902-7 (PMC4780134; doi:10.1186/s13059-016-0902-7)
Supplement: Supplementary file 14 — Supplementary material and methods. (PDF 130 kb) [file 13059_2016_902_MOESM14_ESM.pdf]

Comparative transcriptomics reveals the conserved building blocks involved in parallel evolution of ant phenotypic traits

Authors: Claire Morandin, Mandy M.Y. Tin, Sílvia Abril, Crisanto Gómez, Luigi Pontieri, Morten Schiøtt, Liselotte Sundström, Kazuki Tsuji, Jes Søe Pedersen, Heikki Helanterä, Alexander S. Mikheyev

Supplementary Information

Supplementary Material and Methods

Sample collection. Mature adult worker and queen samples were collected directly in the field from 16 ant species (including two social forms of *Solenopsis invicta*) (Figure 1), right before or during the egg laying period. Seven species of *Formica* ants *(F. aquilonia, F. cinerea, F. exsecta, F. fusca, F. pratensis, F. pressilabris, F. truncorum)* were collected directly from field colonies around the Tvärminne zoological station in Hanko and the Hanko peninsula, southwestern Finland in April 2011, 2012. *Lasius neglectus* samples were collected in May 2012 at Gif sur Yvette (France), and the highland form of *Lasius turcicus* was collected in the fall 2012 near Sandıklı, Afyonkarahisar Province in Turkey, however no queens were found and only workers were used for this study. *Myrmica sulcinodis* and *M. rubra* were sampled in August 2012 on the islands Læsø and Bornholm (Denmark). An additional *Myrmica* species, *M. ruginodis,* was collected in spring 2012 around the Tvärminne zoological station in Hanko (Finland). *Monomorium pharaonis* samples were raised in laboratory in Copenhagen (CSE), and *Monomorium chinense* samples were collected in November 2012 in Okinawa (Japan). Samples of *Linepithema humile* were obtained from Catalonia (Spain) in August 2012. Two social forms

of *Solenopsis invicta*, monogynous and polygynous, were collected in September 2012 in Texas (USA). After collection, samples were brought back to the labs and frozen immediately in –80 °C for later RNA extractions.

For each species, we selected biologically relevant traits of different types including worker sterility (anatomical/physiological for individuals with complete worker sterility (loss of ovaries) being highly derived), colony queen number (a social, colony-level trait with monogyny being primitive and polygyny being derived) and invasiveness (an ecological trait dependent on interactions with other species, where being invasive is derived). Biological traits were deducted from the species biology and previous literature ([1–3], Figure 1).

RNA extractions. Three independent replicates for queens and workers, using whole-body, were used in this study and to obtain sufficient amount of RNA, variable number of samples were pooled per species (see Additional file 1 for more details on pools/libraries composition). The total RNA was extracted using a Trisure protocol for the seven *Formica* species, RNeasy® micro and mini kit (QIAGEN) for the remaining species. Concentrations and qualities of the extracted RNA were examined by Agilent 2100 bioanalyzer (Agilent Technologies). The total amount of input RNA was standardized to 100 ng prior to cDNA synthesis. To control for external variability in the RNA quality, we used RNA spike in [4]. Two different mixes of (ERCC) RNA spike-in control mix were added (1:1000) to assorted RNA samples before cDNA synthesis (an equal number of mix 1 and 2 were added to each species).

All but one species were treated following the procedure described above (for more details on *F. exsecta* extractions and library preparations see [5]). To guarantee that methodological differences did not affect our co-expression network analysis, we used WGCNA cleaning pre-steps to ensure that the *F. exsecta* libraries were not considered as outliers.

cDNA preparation and library preparation

First strand cDNA synthesis. To the RNA and spike-in mix, 2 µL of 10 µM poly T_START oligo5’-AATTGCAGTGGTATCAACGCAGAGCGGCCGCTTTTTTT-TTTTTTTTTTTTTTTTTTTTTTVN were added. The 9 µL mixture was incubated at 65 °C for 3 minutes, and then chilled on ice. The 11 µL reaction mixture containing 4 µL of 5x first strand synthesis buffer (Invitrogen), 1 µL of 10 mM dNTP (Promega), 2 µL of 0.1 M DTT (Invitrogen), 2 µL of 12 µM template switching RNA oligo (5’-AAGCAGUGGUAUCAACGCAGAGUACAUGGG), 1 µLRNase inhibitor (Qiagen) and 1 µL superscript II reverse transcriptase (Invitrogen) were added to each sample. The reactions were incubated at 42 °C for 60 minutes and the enzyme was heat inactivated at 65 °C for 15 minutes. 80 µL MilliQ water were added to each cDNA reaction.

Second strand cDNA synthesis. Second strand cDNA was synthesized with a limited number of PCR cycles. The 50 µL PCR reaction consisted of 1x Phusion HF buffer (Thermo Scientific), 200 µM dNTP (Promega), 0.5 µM START primer (5’-CGCCAGGGTTTTCCCAGTCACGACAATTGCAGTGGTATCAACGCAGA), 0.5 µM TS_long primer (5’-CTTGTAGGTTAAGTGGAGAGCTAACAATTT-CACACAGGAAAGCAGTGGTATCAACGC), 0.5 µL of 2 U/µL Phusion DNA polymerase (Thermo Scientific) and 10 µL diluted cDNA. 50 µL PCR reactions were set up for each cDNA sample. PCR was carried out with the following conditions: initial denaturation at 98 °C for 30 seconds, with 20 cycles of denaturation at 98 °C for 10 seconds, 68 °C for 6 min, followed by final extension at 72° C for 10 min. PCR products were purified by solid phase reversible

immobilization [6] using Dynabeads MyOne Carboxylic Acid (Invitrogen), 17 % PEG was used for purification. The concentration of the DNA was measured with Quant-iT PicoGreen dsDNA Assay Kit (Invitrogen).

Nextera XT library preparation and sequencing. 1 ng of ds-cDNA was used for library preparation using a Nextera XT DNA sample kit (Illumina) according to manufacturer's directions. Eighteen cycles of PCR were used for library amplification. The libraries were size selected before sequencing. In the first selection step, 100 µl of 13 % PEG-6000/NaCl/Tris and 10 µl prepared Dynabeads were added to the library and resuspended. The mixture was incubated for 5 minutes. The tube was then placed on a magnetic stand for 5 minutes. 150 µl supernatant were transferred to a new tube while the beads were discarded. In the second selection, 100 µl of 13.5 % PEG-6000/NaCl/Tris and 10 µl prepared Dynabeads were added to the supernatant and mixed. The mixture was incubated for 5 minutes followed by bead separation on the magnetic stand. This time, the supernatant was discarded and the beads were saved. The beads were washed twice with 70 % ethanol (with 10 mM Tris, pH 6) and dried for 5 minutes. The tubes were then taken off the magnetic stand and DNA was eluted from the beads by resuspending them in 15 µl EB. After 5 minutes incubation, beads were separated from the DNA solution on the magnetic stand. The eluent contained the purified library with peak size of around 380 bp. The libraries were analyzed with Bioanalyzer High Sensitivity DNA Kit (Agilent Technologies). The quantity of the library was estimated by Quant-iT PicoGreen dsDNA Assay Kit and eqimolar of libraries was pooled. Quantitative PCR (KAPA Biosystems) was used to estimate the concentration of the libraries. The pooled library was sequenced paired-end for 100 cycles on an Illumina Hiseq 2000 system at the Okinawa Institute of Science and Technology.

Quality control and validation of RNA-seq data. A multidimensional scaling (MDS) plot was used to illustrate the overall similarity of expression profiles across samples/libraries. RNA spike-in libraries were used to determine the dynamic range of our experiment and to provide a sensible cutoff for data filtration during differential expression analysis. Each spike-in is a set of unlabeled, polyadenylated transcripts, available in two mixes with different concentration, and are a mean to control for external variability in the RNA quality [4]. These transcripts were used to evaluate the dynamic range of the experiment and its sensitivity to detect changes in transcript abundance (ERCC). Two different mixes of (ERCC) RNA spike-in control mix were added (1:1000) to assorted RNA samples before cDNA synthesis. We then used the strength of expression between expected and observed spike-in levels to determine the optimal level of FPKM abundance cutoff.

Module preservation. We also conducted module preservation statistics using WGCNA modules retrieved from a recent study of worker behavior [31, 77]. We compared the extent of module preservation in an independent data set by checking whether there was correspondence in module assignment between this study and an earlier study of behavioral polyethism in *Monomorium pharaonis*, which also used WGCNA [31]. Orthologs of *M. pharaonis* genes were selected using BLAST. We then calculated how often genes were classified as belonging to the same module by both studies [77]. Statistical significance was determined using Fisher's exact test, adjusted for multiple comparisons using FDR with the false discovery rate set at 0.05.

Supplementary Results

Caste-biased genes. The number of differentially expressed genes between queen and worker varied among species (e.g. between 323 genes (7.4 % of the total number of genes kept for the analysis) in *Formica aquilonia* to 3834 genes (84 %) in *Solenopsis invicta* monogynous form and 5502 genes (72%) in *Linepithema humile* (FDR corrected $p < 0.05$)). All but four species (*Formica pressilabris* (2049 queen-biased genes, 1633 worker-biased genes)*, Linepithema humile* (2832, 2670), *Myrmica ruginodis* (1502, 1476) and *Myrmica sulcinodis* (1263, 1191)) presented a higher number of worker upregulated genes compared to the queen. In total, 21,465 worker genes and 18,218 queen genes were found to be caste-biased.

Module functional annotation. Among the queen-related pathways we identified modules linked to cellular responses and regulations (module 5), signal transduction and DNA damage check (module 6), cuticle and amino acid processes (module 13), healing and nucleic acid processes (module 20), protein signal transduction and regulation of biological processes (module 21), Ras signaling (module 27), metabolic processes (module 28), cellular component organization and cell division (module 31) and protein fidelity and catabolism (module 34). Among the worker-related module pathways we identified GO terms linked to circadian cycle and behavior (module 7), protein and metabolic processes (module 8), sensory perception (module 10), signal transduction (module 12), metabolic processes (module 17), biosynthesis and metabolism (module 25), redox and apoptosis (module 26), gene expression and translation (module 32), transmembrane transport and biosynthetic processes (module 33) and protein targeting (module 36). Full list of GO terms can be found in Additional file 8.

d_N/d_S and connectivity of phenotypic-associated genes.

Worker sterility – d_N/d_S values were not significantly different between sterile- and non traits-associated genes (NTA) (GLM, $p = 0.7$). Worker sterility-associated genes had significantly lower connectivity than non traits-associated genes (GLM, $p = < 0.001$). Worker sterility associated genes had significantly lower expression levels than non traits-associated genes (GLM, $p < 0.001$).

Queen number – d_N/d_S values were not significantly different between single queen-associated genes and non traits-associated genes (GLM, $p = 0.41$). Single queen- connectivity values were significantly lower than non traits-associated values (GLM, $p < 0.01$). Single queen-associated genes had significantly lower expression levels than non traits-associated genes (GLM, $p < 0.001$).

Invasiveness – No differences in the rates of evolution could be detected between non invasiveness- and non traits-associated genes (GLM, $p = 0.08$). Connectivity was significantly higher for non traits-associated-genes compared to non invasiveness-associated genes (GLM, $p = 0.03$). Expression levels of non invasiveness-associated genes were significantly lower than non traits-associated genes (GLM, $p < 0.001$).

References

1. Helanterä H, Sundström L: **Worker Reproduction in *Formica* ants.** *Am Nat* 2007, **170**:E14–E25.

2. Sundström L, Seppä P, Pamilo P: **Genetic population structure and dispersal patterns in *Formica* ants — a review**. *Ann Zool Fennici* 2005, **42**:163–177.

3. Espadaler X, Rey S: **Biological constraints and colony founding in the polygynous invasive ant *Lasius neglectus* (Hymenoptera, Formicidae)**. *Insectes Soc* 2001, **48**:159–164.

4. Jiang L, Schlesinger F, Davis CA, Zhang Y, Li R, Salit M, Gingeras TR, Oliver B: **Synthetic spike-in standards for RNA-seq experiments.** *Genome Res* 2011, **21**:1543–51.

5. Morandin C, Dhaygude K, Paviala J, Trontti K, Wheat C, Helanterä H: **Caste-biases in gene expression are specific to developmental stage in the ant *Formica exsecta***. *J Evol Biol* 2015.

6. Tin MMY, Rheindt FE, Cros E, Mikheyev AS: **Degenerate adaptor sequences for detecting PCR duplicates in reduced representation sequencing data improve genotype-calling accuracy.** *Mol Ecol Resour* 2014, **15**:329–336.
