# Supplementary material for: Comparative transcriptomics reveals the conserved building blocks involved in parallel evolution of diverse phenotypic traits in ants
Source: Genome Biol. 2016 Mar 7;17:43. doi: 10.1186/s13059-016-0902-7 (PMC4780134; doi:10.1186/s13059-016-0902-7)
Supplement: Supplementary file 19 — Scale free topology criterion with a R^2 threshold of 0.9. A soft threshold power of 8 was chosen. (PDF 44 kb) [file 13059_2016_902_MOESM19_ESM.pdf]

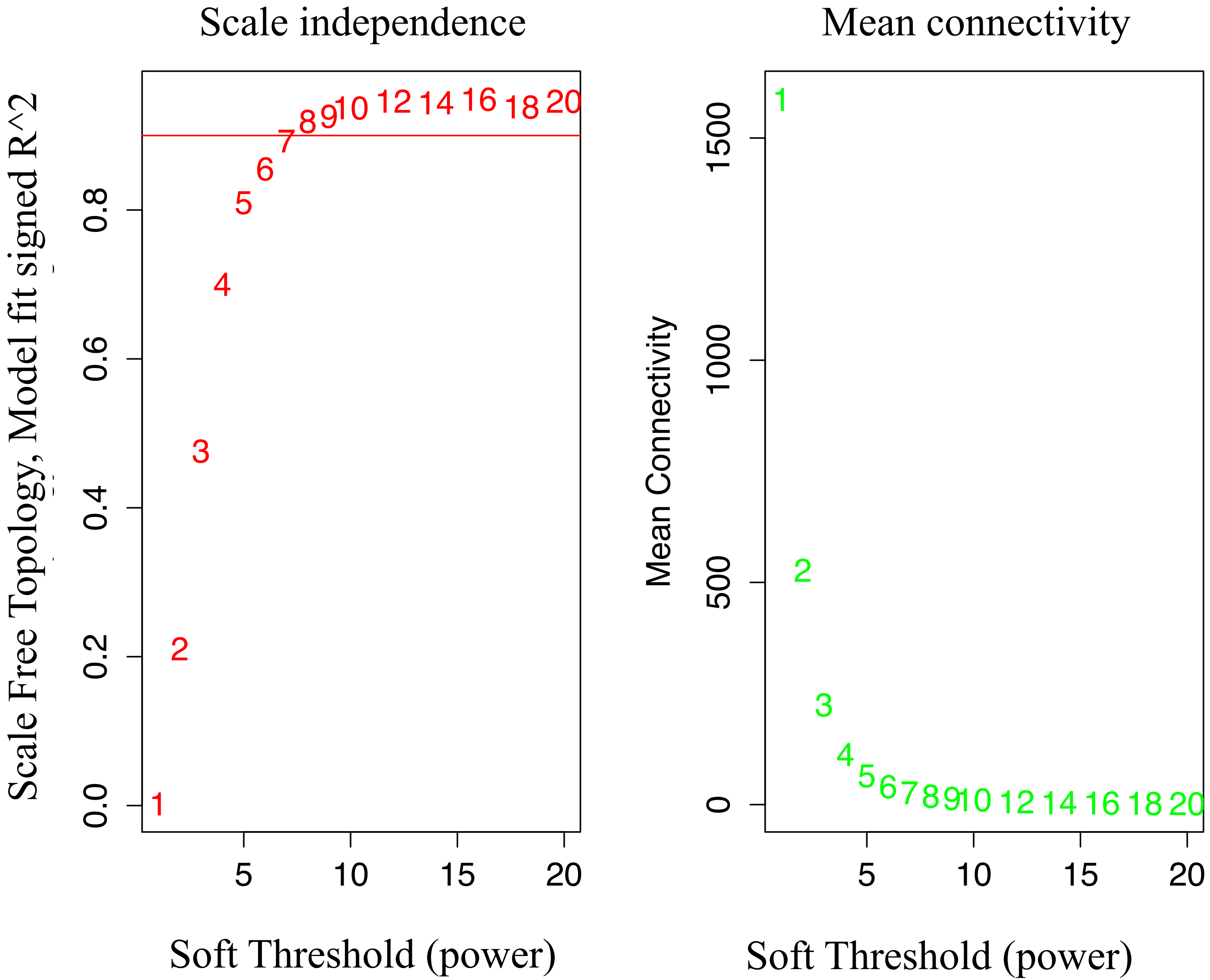

Scale independence
Scale Free Topology, Model fit signed R^2
Soft Threshold (power)
Mean connectivity
Mean Connectivity
Soft Threshold (power)
