# Supplementary material for: Comparative transcriptomics reveals the conserved building blocks involved in parallel evolution of diverse phenotypic traits in ants
Source: Genome Biol. 2016 Mar 7;17:43. doi: 10.1186/s13059-016-0902-7 (PMC4780134; doi:10.1186/s13059-016-0902-7)
Supplement: Supplementary file 22 — Detailed workflow of the WGCNA analysis. (PDF 520 kb) [file 13059_2016_902_MOESM22_ESM.pdf]

Detailed workflow for WGCNA analysis for Morandin et al. Comparative transcriptomics reveals the conserved building blocks involved in parallel evolution of diverse phenotypic traits in ants

```
library(flashClust)
library(WGCNA)
library(dynamicTreeCut)
library(Hmisc)
```

#PART 1: Loading and Cleaning the Data

```
library(WGCNA)
library(dynamicTreeCut)
options(stringsAsFactors = FALSE);

dim(dat1)
names(dat1)
datExpr0 = as.data.frame(t(dat1[, -c(1)]));
names(datExpr0) = dat1$cluster;
rownames(datExpr0) = names(dat1)[-c(1)];

#Check genes with too many missing values
gsg = goodSamplesGenes(datExpr0, verbose = 3)
gsg$allOK

if (!gsg$allOK)
{
if (sum(!gsg$goodGenes)>0)
   printFlush(paste("Removing genes:", paste(names(datExpr0)[!gsg$goodGenes],
collapse = ", ")));
  if (sum(!gsg$goodSamples)>0)
   printFlush(paste("Removing samples:",
paste(rownames(datExpr0)[!gsg$goodSamples], collapse = ", ")));
datExpr0 = datExpr0[gsg$goodSamples, gsg$goodGenes]
}

#Cluster the samples to inspect for outlier arrays.
sampleTree = flashClust(dist(datExpr0), method = "average");
sizeGrWindow(12,9)
par(cex = 0.6);
par(mar = c(0,4,2,0))
plot(sampleTree, main = "Sample clustering to detect outliers", sub="", xlab="",
cex.lab = 1.5,cex.axis = 1.5, cex.main = 2)
abline(h = 200000, col = "red");
clust = cutreeStatic(sampleTree, cutHeight = 300000, minSize = 10)
```

```
table(clust)
```

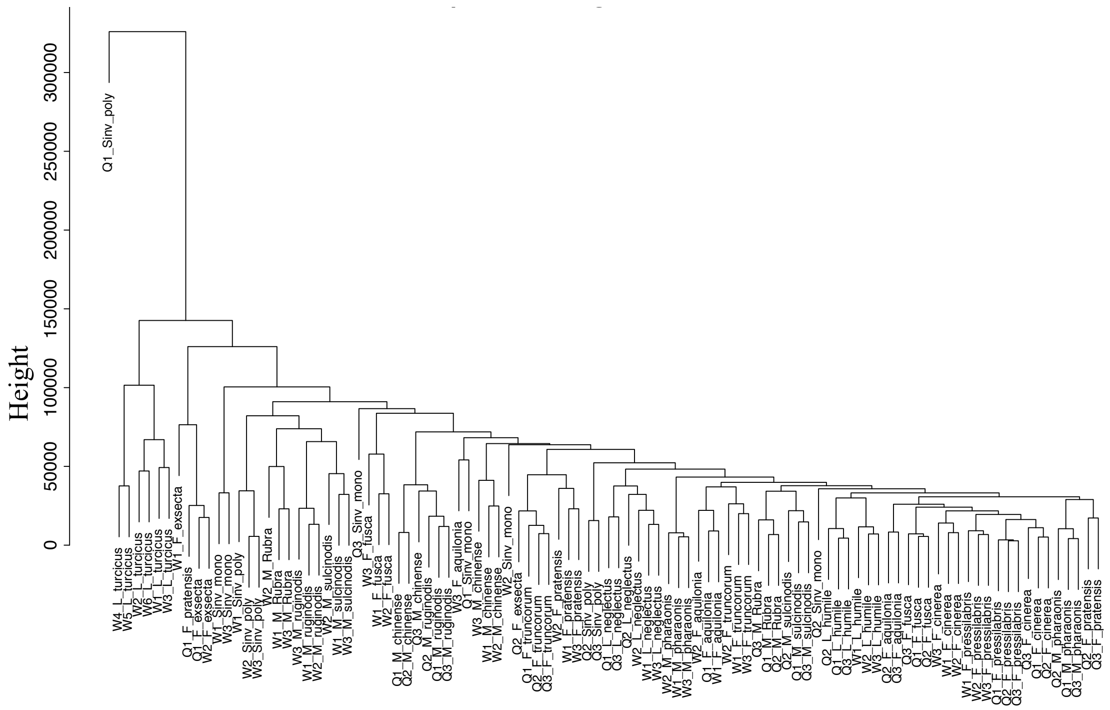


```
keepSamples = (clust==1)
datExpr = datExpr0[keepSamples, ]
nGenes = ncol(datExpr)
nSamples = nrow(datExpr)

traitData = read.csv("traits.csv", header=T);
dim(traitData)
names(traitData)
allTraits = traitData;
dim(allTraits)
names(allTraits)
WWDSDRSamples = rownames(datExpr);
traitRows = match(WWDSDRSamples, allTraits$Samples);
datTraits = allTraits[traitRows, -1];
rownames(datTraits) = allTraits[traitRows, 1];
collectGarbage();
sampleTree2 = flashClust(dist(datExpr), method = "average")
traitColors = numbers2colors(datTraits, signed = FALSE);
```

#PART2: Network Construction and Module Detection

```
powers = c(c(1:10), seq(from = 12, to=20, by=2))

#Call the network topology analysis function
sft = pickSoftThreshold(datExpr, powerVector = powers, verbose = 5)
```

```
pickSoftThreshold: will use block size 7427.
 pickSoftThreshold: calculating connectivity for given powers...
   ..working on genes 1 through 7427 of 7427
```

Power	SFT.R.sq	slope	truncated.R.sq	mean.k.	median.k.	max.k.
1	0.0024	-0.307	0.974	1590.00	1580.000	2540.0
2	0.2110	-1.820	0.956	528.00	520.000	1220.0
3	0.4760	-2.380	0.937	224.00	217.000	710.0
4	0.7000	-2.880	0.966	113.00	105.000	472.0
5	0.8090	-3.000	0.982	64.10	57.300	340.0
6	0.8550	-3.010	0.992	40.00	33.700	259.0
7	0.8930	-2.820	0.989	26.70	21.100	205.0
8	0.9190	-2.660	0.989	18.80	13.800	167.0
9	0.9260	-2.570	0.983	13.80	9.450	145.0
10	0.9370	-2.510	0.994	10.50	6.640	129.0
12	0.9460	-2.390	0.997	6.56	3.540	105.0
14	0.9440	-2.290	0.991	4.42	2.050	88.1
16	0.9490	-2.210	0.987	3.15	1.250	76.9
18	0.9380	-2.170	0.977	2.34	0.787	68.1
20	0.9460	-2.090	0.981	1.80	0.519	61.0

```
sizeGrWindow(9, 5)
par(mfrow = c(1,2));
cex1 = 1;
plot(sft$fitIndices[,1], -sign(sft$fitIndices[,3])*sft$fitIndices[,2], xlab="Soft
Threshold (power)",ylab="Scale Free Topology Model Fit, signed R^2", type="n",
main = paste("Scale independence"));
text(sft$fitIndices[,1], -sign(sft$fitIndices[,3])*sft$fitIndices[,2],
labels=powers,cex=cex1,col="red");
abline(h=0.90,col="red")
plot(sft$fitIndices[,1], sft$fitIndices[,5], xlab="Soft Threshold (power)",ylab="Mean
Connectivity", type="n", main = paste("Mean connectivity"))
text(sft$fitIndices[,1], sft$fitIndices[,5], labels=powers, cex.axis =
3,col="green",cex.lab =20,cex.main =2)
```

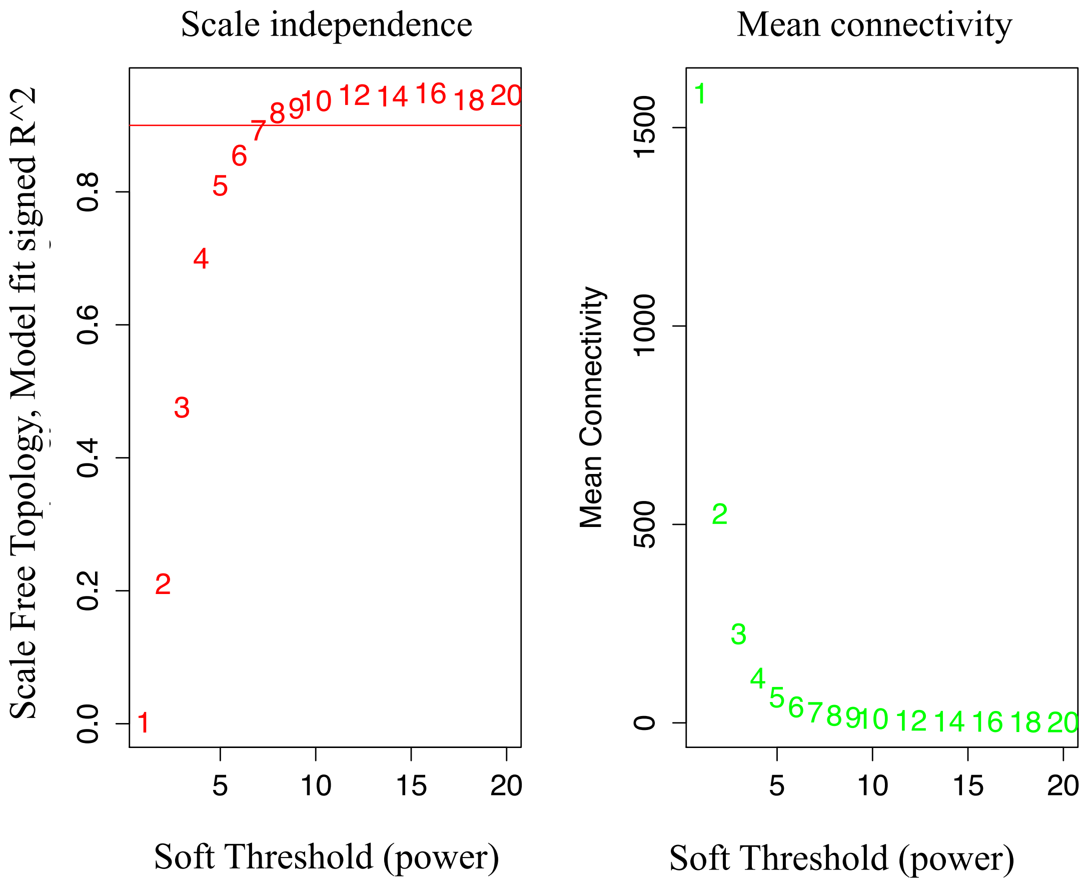


```
#Now calculate the adjacencies using the soft thresholding power (i.e., softPower =
beta)
softPower = 8;
adjacency = adjacency(datExpr, power = softPower);

#Turn adjacency matrix into topological overlap matrix; then convert to dissimilarity
matrix (1-TOM).
TOM = TOMsimilarity(adjacency);
dissTOM = 1-TOM
geneTree = flashClust(as.dist(dissTOM), method = "average");

#Module identification using dynamic tree cut
minModuleSize = 30;
dynamicMods = cutreeDynamic(dendro = geneTree, distM = dissTOM, deepSplit = 2,
cutHeight = 0.97, pamRespectsDendro = FALSE, minClusterSize = minModuleSize);
table(dynamicMods)
dynamicColors = labels2colors(dynamicMods)
table(dynamicColors)

#Calculate eigengenes
MEList = moduleEigengenes(datExpr, colors = dynamicColors)
MEs = MEList$eigengenes
#Calculate dissimilarity of module eigengenes
```

```
MEDiss = 1-cor(MEs);

#Cluster module eigengenes
METree = flashClust(as.dist(MEDiss), method = "average");

#Plot the result
sizeGrWindow(7, 6)
plot(METree, main = "Clustering of module eigengenes", xlab = "", sub = "")
MEDissThres = 0.2
abline(h=MEDissThres, col = "red")
```

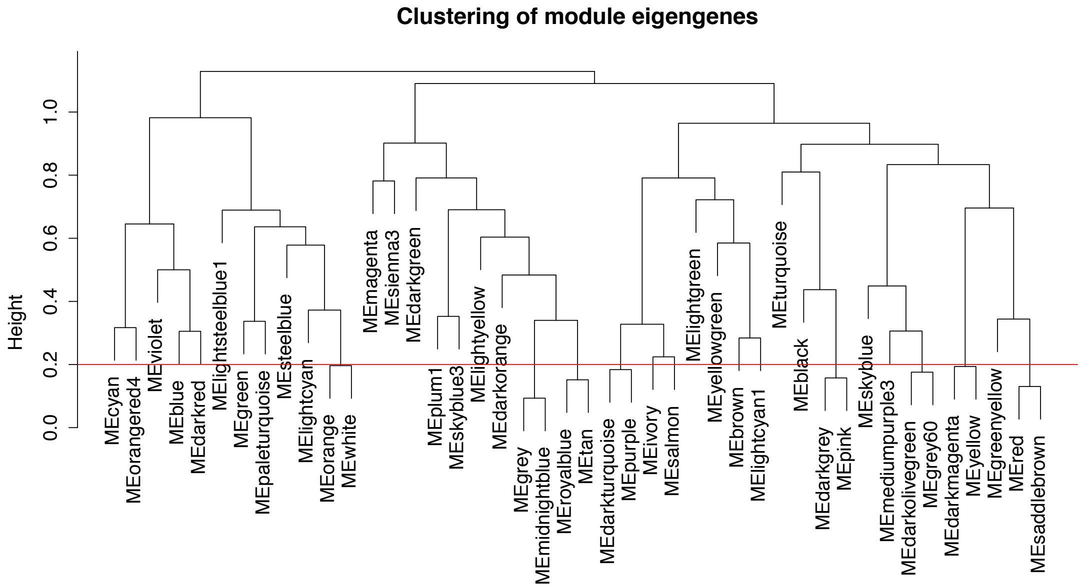


```
#Call automatic merging function
merge = mergeCloseModules(datExpr, dynamicColors, cutHeight = MEDissThres,
verbose = 3)
mergedColors = merge$colors;
mergedMEs = merge$newMEs;
```

```
mergeCloseModules: Merging modules whose distance is less than 0.2
  multiSetMEs: Calculating module MEs.
    Working on set 1 ...
    moduleEigengenes: Calculating 44 module eigengenes in given set.
  multiSetMEs: Calculating module MEs.
    Working on set 1 ...
    moduleEigengenes: Calculating 37 module eigengenes in given set.
  Calculating new MEs...
  multiSetMEs: Calculating module MEs.
    Working on set 1 ...
    moduleEigengenes: Calculating 37 module eigengenes in given set.
```

```
sizeGrWindow(12, 9)
```

```
plotDendroAndColors(geneTree, cbind(dynamicColors, mergedColors), c("Dynamic Tree Cut", "Merged dynamic"),
                    dendroLabels = FALSE, hang = 0.03, addGuide = TRUE, guideHang = 0.05)
```

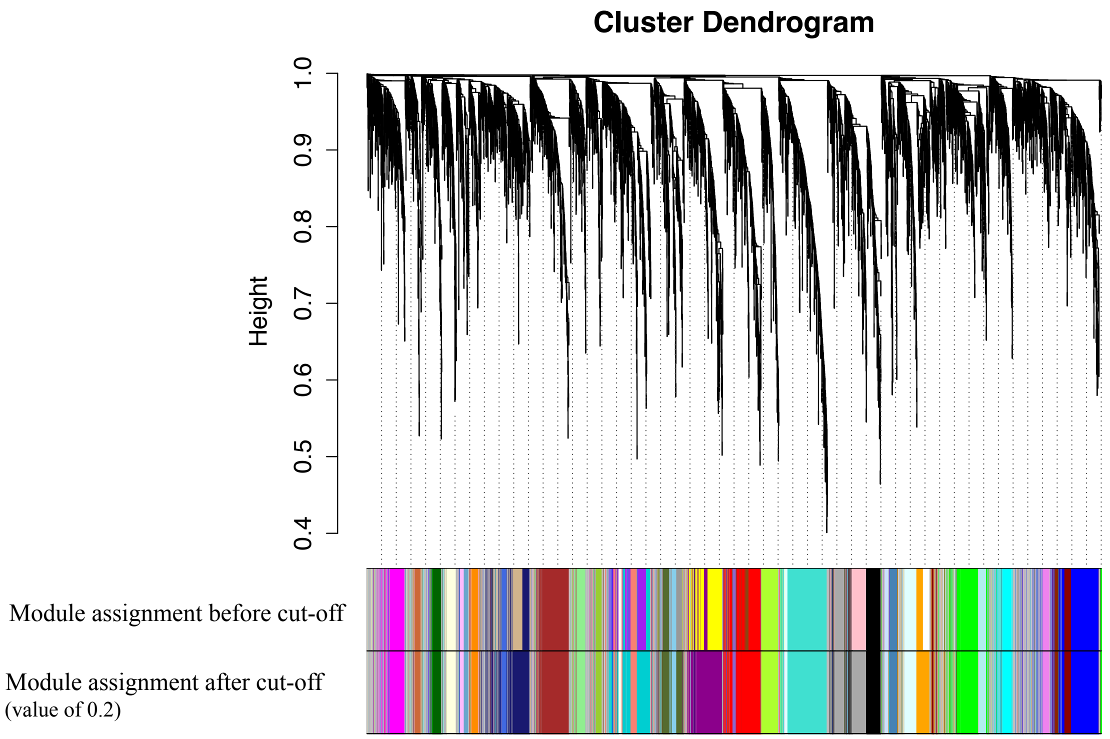


```
moduleColors = mergedColors
colorOrder = c("grey", standardColors(50));
moduleLabels = match(moduleColors, colorOrder)-1;
MEs = mergedMEs;
```

#PART3: Related modules to external information and identifying important genes

```
# Define numbers of genes and samples
nGenes = ncol(datExpr);
nSamples = nrow(datExpr);

#Recalculate MEs with color labels
MEs0 = moduleEigengenes(datExpr, moduleColors)$eigengenes
MEs = orderMEs(MEs0)
moduleTraitCor = cor(MEs, datTraits, method="pearson");
moduleTraitPvalue = corPvalueStudent(moduleTraitCor, nSamples);
moduleTraitCor=cor(MEs, datTraits, method="pearson");

#MCMCglmm analysis (For subsequent analysis, Additional file 21 was used to
correlate modules eigengenes with external traits)

library(MCMCglmm)

#Data
phylo1<- read.tree(file="tree_mcmc.tre")

data = read.table("TableS8.txt", header =T)
attach(data)

#Ultrametric tree
chronopl(phylo1, lambda=0.1) -> phylo
inv.phylo<-inverseA(phylo,nodes="TIPS")
prior<-list(R=list(V=1, nu=0.002), G=list(G1=list(V=1, nu=0.002)))

#Module Magenta
model1<-MCMCglmm(ME1 ~ Caste + Worker sterility + Queen number +
Invasiveness ,random=~phylo,
ginverse=list(phylo=inv.phylo$Ainv),prior=prior,data=data,nitt=500000,burnin=1000
00,thin=500,verbose = FALSE)

model2<-MCMCglmm(ME1 ~ Caste + Worker sterility + Queen number +
Invasiveness ,random=~phylo,
ginverse=list(phylo=inv.phylo$Ainv),prior=prior,data=data,nitt=500000,burnin=1000
00,thin=500,verbose = FALSE)

model3<-MCMCglmm(ME1 ~ Caste + Worker sterility + Queen number +
Invasiveness ,random=~phylo,
ginverse=list(phylo=inv.phylo$Ainv),prior=prior,data=data,nitt=500000,burnin=1000
00,thin=500,verbose = FALSE)

chainList_DG <- mcmc.list(model1$Sol,model2$Sol,model3$Sol)
gelman.diag(chainList_DG)
plot(chainList_DG)
summary(chainList_DG)
```

Repeat for all modules
